# Symposium Mammographicum 2023

**DOI:** 10.1186/s13058-023-01702-8

**Published:** 2023-10-10

## W7.1 Can axillary surgery be tailored based on response to neoadjuvant chemotherapy assessed with CEUS?

### Mariana Afonso Matias^1^; Nisha Sharma^1^; Isobel Haigh^1^; Rebecca Millican-Slater^1^; Raj Achuthan^1^; Yan Chen^2^

#### ^1^Leeds Teaching Hospital NHS Trust; ^2^University of Nottingham

##### ***Correspondence***: Mariana Afonso Matias

***Breast Cancer Research*** 2023,** 25 (Suppl 2)**:W7.1

**Background:** This feasibility study was designed to evaluate if contrast-enhanced ultrasound (CEUS) can identify node-positive patients before neoadjuvant chemotherapy (NACT) and assess the residual cancer burden within the axilla following NACT and therefore, potentially tailor the surgical treatment of the axilla.

**Methods:** 32 patients were identified of which 26 met the inclusion criteria and underwent CEUS pre and post NACT. The sentinel lymph nodes (SLNs) identified during CEUS were biopsied and clipped. All 26 participants of this study underwent axillary node clearance (ANC) along with breast conservative surgery or mastectomy. Axillary specimens were further reviewed considering the number of positive nodes and if nodes clipped during CEUS were positive or negative.

**Results:** Following NACT, among the 26 participants, CEUS identified positive SLNs in 8 patients. Post ANC, histology showed that 7/8 of these patients had positive nodes. The remaining 18 patients were found to be node negative on CEUS post NACT. Furthermore, post ANC, the histology of 12 of these patients showed positive nodes with a tumour burden ranging from 1 to 18 LNs. Further analysis showed that in 25% of these patients the identified positive nodes were non SLNs.

**Conclusions:** The study shows that although the SLN maybe negative post NACT that non-SLNs may still be malignant and therefore a negative SLN does not translate into a negative axillary clearance. Therefore further studies looking at the role of targeted axillary dissection such as the ATNEC trial are important to support de-escalation of axillary surgery in the context of NACT.

**References**

- Carlson, R. W., Allred, D. C., Anderson, B. O., Burstein, H. J., Carter, W. B., Edge, S. B., Erban, J. K., Farrar, W. B., Goldstein, L. J., Gradishar, W. J., Hayes, D. F., Hudis, C. A., Jahanzeb, M., Kiel, K., Ljung, B. M., Marcom, P. K., Mayer, I. A., McCormick, B., Nabell, L. M., Wolff, A. C. (2009). Breast Cancer. *Journal of the National Comprehensive Cancer Network*, *7*(2), 122–192. 10.6004/jnccn.2009.0012
- Cortazar, P., Zhang, L., Untch, M., Mehta, K., Costantino, J. P., Wolmark, N., Bonnefoi, H., Cameron, D., Gianni, L., Valagussa, P., Swain, S. M., Prowell, T., Loibl, S., Wickerham, D. L., Bogaerts, J., Baselga, J., Perou, C., Blumenthal, G., Blohmer, J., von Minckwitz, G. (2014). Pathological complete response and long-term clinical benefit in breast cancer: the CTNeoBC pooled analysis. *The Lancet*, *384*(9938), 164–172. 10.1016/s0140-6736(13)62422-8
- Cimmino, V. M., Brown, A. C., Szocik, J. F., Pass, H. A., Moline, S., De, S. K., & Domino, E. F. (2001). Allergic reactions to isosulfan blue during sentinel node biopsy—a common event. *Surgery*, *130*(3), 439–442. 10.1067/msy.2001.116407
- Cui, X., Ignee, A., & Bachmann Nielsen, M. (2013). Contrast enhanced ultrasound of sentinel lymph nodes. *Journal of Ultrasonography*, *13*(52), 73–81. 10.15557/jou.2013.0006
- Dominici, L. S., Negron Gonzalez, V. M., Buzdar, A. U., Lucci, A., Mittendorf, E. A., Le-Petross, H. T., Babiera, G. V., Meric-Bernstam, F., Hunt, K. K., & Kuerer, H. M. (2010). Cytologically proven axillary lymph node metastases are eradicated in patients receiving preoperative chemotherapy with concurrent trastuzumab for HER2-positive breast cancer. *Cancer*, *116*(12), 2884–2889. 10.1002/cncr.25152
- Hennessy, B. T., Hortobagyi, G. N., Rouzier, R., Kuerer, H., Sneige, N., Buzdar, A. U., Kau, S. W., Fornage, B., Sahin, A., Broglio, K., Singletary, S. E., & Valero, V. (2005). Outcome After Pathologic Complete Eradication of Cytologically Proven Breast Cancer Axillary Node Metastases Following Primary Chemotherapy. *Journal of Clinical Oncology*, *23*(36), 9304–9311. 10.1200/jco.2005.02.5023
- King, T. A., & Morrow, M. (2015). Surgical issues in patients with breast cancer receiving neoadjuvant chemotherapy. *Nature Reviews Clinical Oncology*, *12*(6), 335–343. 10.1038/nrclinonc.2015.63
- Liu, J., Liu, X., He, J., Gou, B., Luo, Y., Deng, S., Wen, H., & Zhou, L. (2019). Percutaneous contrast-enhanced ultrasound for localization and diagnosis of sentinel lymph node in early breast cancer. *Scientific Reports*, *9*(1). 10.1038/s41598-019-49736-3
- Luo, J., Feng, L., Zhou, Q., Chen, Q., Liu, J., Wu, C., Luo, J., Chen, J., Wu, H., & Deng, W. (2021). The value of contrast‐enhanced ultrasound in determining the location of sentinel lymph nodes in breast cancer. *Cancer Imaging*, *21*(1). 10.1186/s40644-021-00397-4
- Mansel, R. E., Fallowfield, L., Kissin, M., Goyal, A., Newcombe, R. G., Dixon, J. M., Yiangou, C., Horgan, K., Bundred, N., Monypenny, I., England, D., Sibbering, M., Abdullah, T. I., Barr, L., Chetty, U., Sinnett, D. H., Fleissig, A., Clarke, D., & Ell, P. J. (2006). Randomized Multicenter Trial of Sentinel Node Biopsy Versus Standard Axillary Treatment in Operable Breast Cancer: The ALMANAC Trial. *JNCI: Journal of the National Cancer Institute*, *98*(9), 599–609. 10.1093/jnci/djj158
- Provenzano, E. (2021). Neoadjuvant Chemotherapy for Breast Cancer: Moving Beyond Pathological Complete Response in the Molecular Age. *Acta Medica Academica*, *50*(1), 88. 10.5644/ama2006-124.328
- Sever AR, Mills P, Jones SE, Cox K, Weeks J, Fish D et al. (2011). Preoperative sentinel node identification with ultrasound using microbubbles in patients with breast cancer. *AJR Am J Roentgenol*, 196: 251–256.

## W7.2 Mammographic density and breast cancer risk factors: Systematic review and meta-analysis

### Dixa Thakrar; Zoe Grenville; Trishna Desai; Toral Gathani; Gillian K Reeves; Isobel Barnes

#### Cancer Epidemiology Unit, Nuffield Department of Population Health, University of Oxford

##### ***Correspondence***: Dixa Thakrar

***Breast Cancer Research*** 2023, **25 (Suppl 2)**:W7.2

**Background:** Mammographic density (MD) is an important risk factor for breast cancer and reduces mammographic screening sensitivity (1,2). There are several breast cancer risk factors which have been consistently shown to be associated with MD, including age, menopausal status, BMI, and menopausal hormone therapy (3–9). However, the associations of some lifestyle and reproductive factors with MD are uncertain. We conducted a systematic review to examine the associations of alcohol, smoking, parity, age at first birth and age at menarche, with MD.

**Methods:** Articles published between 2000 and 2021, reporting the associations of the risk factors of interest with age-adjusted MD, were identified. Study quality was assessed using tools from the Joanna Briggs Institute. Fixed-effects meta-analyses were conducted to synthesise the results.

**Results:** Fifty-nine studies were included. Pooled results suggested that increased alcohol intake, and later age at first birth and menarche were associated with increased MD, while increased parity and smoking were associated with decreased MD: e.g. odds ratio (OR) for higher MD in alcohol drinkers versus non-drinkers was 1.15 (95%CI 1.06–1.25; p = 0.001) and OR for parous versus nulliparous women was 0.68 (0.65–0.71; p < 0.001). There was significant statistical heterogeneity among studies.

**Conclusions:** There is some evidence that lifestyle and reproductive risk factors are associated with MD. Several studies were small with heterogeneous results, limiting reliable interpretation of the findings. Further work using data from a large prospective study is planned, to reliably investigate how these risk factors influence screening sensitivity and breast cancer risk, through their effects on MD.

**References**

1. McCormack VA, Dos Santos Silva I. Breast density and parenchymal patterns as markers of breast cancer risk: A meta-analysis. Cancer Epidemiology Biomarkers and Prevention. 2006;15(6):1159–69.
2. Weigel S, Heindel W, Heidrich J, Hense HW, Heidinger O. Digital mammography screening: sensitivity of the programme dependent on breast density. Eur Radiol. 2017 Jul 1;27(7):2744–51.
3. Azam S, Jacobsen KK, Aro AR, Lynge E, Andersen ZJ. Hormone replacement therapy and mammographic density: a systematic literature review. Breast Cancer Res Treat [Internet]. 2020;182(3):555–79. Available from: 10.1007/s10549-020-05744-w
4. Checka CM, Chun JE, Schnabel FR, Lee J, Toth H. The relationship of mammographic density and age: Implications for breast cancer screening. American Journal of Roentgenology. 2012;198(3):292–5.
5. Burton A, Maskarinec G, Perez-Gomez B, Vachon C, Miao H, Lajous M, et al. Mammographic density and ageing: A collaborative pooled analysis of cross-sectional data from 22 countries worldwide. PLoS Med. 2017;14(6):1–20.
6. Moore JX, Han Y, Appleton C, Colditz G, Toriola AT. Determinants of Mammographic Breast Density by Race Among a Large Screening Population. JNCI Cancer Spectr. 2020;0:1–10.
7. Hart V, Reeves KW, Sturgeon SR, Reich NG, Sievert LL, Kerlikowske K, et al. The effect of change in body mass index on volumetric measures of mammographic density. Cancer Epidemiology Biomarkers and Prevention. 2015;24(11):1724–30.
8. Sprague BL, Gangnon RE, Burt V, Trentham-Dietz A, Hampton JM, Wellman RD, et al. Prevalence of mammographically dense breasts in the United States. J Natl Cancer Inst [Internet]. 2014 Oct;106(10). Available from: http://www.ncbi.nlm.nih.gov/pubmed/25217577
9. Titus-Ernstoff L, Tosteson ANA, Kasales C, Weiss J, Goodrich M, Hatch EE, et al. Breast cancer risk factors in relation to breast density (United States). Cancer Causes and Control. 2006;17(10):1281–90.

## W7.3 Retrospective audit of patients with breast pain referred to the Bristol breast care centre

### Kanika Kaushal^1^; Alexandra Valencia^2^

#### ^1^Royal Liverpool University Hospital; ^2^North Bristol NHS Trust

##### ***Correspondence***: Kanika Kaushal

***Breast Cancer Research*** 2023, **25 (Suppl 2)**:W7.3

**Objectives:** A retrospective audit at the Bristol breast care centre was performed to

- Establish the incidence of breast cancer in patients referred with breast pain and whether patients diagnosed with cancer had other symptoms
- Assess P value for the clinical exam
- Establish cancer visibility on mammography

**Methods:** Review of Imaging and clinical notes of 26/2952 women referred with breast pain from July 2020 to June 2021 and diagnosed with breast cancer.

**Results:**

- 17/24 cancers were diagnosed in the area of concern, 7/24 were incidental, 15/17 cancers in women over the age of 40
- 12/17 patients had pain and lump at the site of clinical concern, 1/17 had pain and nipple inversion, 1/17 had lump only, 3/17 had pain only
- P values for clinical exams were as follows:
  - GP 10/17 patients lump felt (59%), 6/17 (35%) referred as suspicious
  - Surgeon/NP—15/17 patients (88%), 3/17- P2, 4/17- P3, 8/17 (47%)- P4/5
- 13/17 (76%) MXR in women over 40 reported as M4/5
- 3/2952 (0.1%) patients referred with breast pain only were diagnosed with cancer in the area of concern
- 7/2952 (0.2%) had incidental cancers

**Conclusion:**

- Most women referred with breast pain to the one-stop clinic did not have a diagnosis of cancer
- 24/2952 (0.81%) cases referred for breast pain had breast cancer
- Just 3/2952 (0.1%) had breast pain only at diagnosis
- Most women diagnosed with cancer had an additional symptom, mostly breast lumps
- Most cancers were seen on mammography, which was more specific than the clinical examination P value

## W7.4 Breast lesions incidentally detected on CT- audit of cases referred to a one stop breast clinic

### Olivia Taylor-Fry; Shahrooz Mohammadi

#### St Georges NHS Trust

##### ***Correspondence***: Olivia Taylor-Fry

***Breast Cancer Research*** 2023, **25 (Suppl 2)**:W7.4

**Background:** The increasing use of cross sectional imaging has led to more incidental breast lesions being detected. An increasing number of patients are therefore referred to breast clinic following incidental findings on CT.

**Objective:** To look at the referral pathway following detection of a breast abnormality on CT, outcome of assessment and waiting times.

**Method:** Retrospective search of RIS (March-Nov 2020): all CT referrals that included chest imaging were included. Those that had subsequent specific breast imaging were reviewed, and all CT reports were assessed to see if reference was made to breast investigation or breast lesions to determine whether the CT/Radiology had initiated the breast referral.

Data collected: patient demographics, radiological findings, time to be seen in breast clinic, imaging characteristics of the CT findings, dedicated breast imaging findings, and final pathology.

**Results:**

3535 CT examinations performed from March to Nov 2020.

78 referred to the Breast Unit. 36% (n = 28) had biopsies, 64% of cases (n = 50) no biopsies were required.

17% of the CT referred incidental breast lesions were malignant.

Referral type: 38/78 referrals advised correct referral pathway via the one stop breast clinic/ triple assessment in the CT report.

14/78 CT reports did not mention specific breast referrals (although breast pathology was mentioned in the CT report).

Waiting times: 26 patients seen in breast one stop within 14 days of CT performed.

30 waited over 28 days.

**Conclusions:** Propose new pathway to involve breast radiologist.

**References**

- Georgieva M. Rennert J. Brochhausen C. Stroszczynski C. Jung EM. (2021) Suspicious breast lesions incidentally detected on chest computed tomography with histopathological correlation. The Breast journal. 27: 715–722
- Hussain A, Gordon-Dixon A, Almusawy H, Sinha P, Desai A. (2010) The incidence and outcome of incidental breast lesions detected by computed tomography. Ann R Coll Surg Engl 2010; 92: 124–126
- Lin W, Hsu H. Li C (2011) Incidentally detected enhancing breast lesions on chest computed tomography. Korean Journal of Radiology. 12:44–45.
- Prionas N. Lindfors K. Ray S (2010) Contrast enhanced dedicated breast CT: initial clinical experience. Radiology 256(3): 714–723
- Moyle P. Sonoda L. Britton P. Sinnatamby R (2010) Incidental breast lesions detected on CT: what is their significance? The British Journal of Radiology 83: 233–240

## W7.5 Incidental breast lesions on cross-sectional studies -impact of an alternated referral pathway BREAST 1 code

### Anusha Subramanian; Richard Sidebottom; Iain Lyburn; Sarah Vinnicombe

#### Thirlestaine Breast Centre Cheltenham

##### ***Correspondence***: Sarah Vinnicombe

***Breast Cancer Research*** 2023, **25 (Suppl 2)**:W7.5

**Background**: Increasing numbers of breast lesions are identified on body cross-sectional studies, resulting in increased referrals to symptomatic Breast clinics, often unnecessary.

An automatic triage system was developed to manage these. Dictation of the code BREAST1 in a report on identification of a breast "incidentaloma" triggers a dedicated email to breast radiology. After review and comparison with prior breast imaging, an addendum is dictated, indicating whether referral is necessary.

**Study Aim**:  To evaluate the impact of the BREAST1 referral pathway on numbers of referrals to the Breast clinic and clinical outcomes.

**Method**:  A retrospective analysis of all referrals with the BREAST1 code between December 2018 and October 2022.

**Results**:  Of 372 instances:

- No action was necessary in 191 patients (51%) with longstanding or benign findings.
- Referral to the Breast clinic was recommended in 165 patients (44%).
  - 27 (16%) were not referred due to comorbidities. 3 were subsequently diagnosed with invasive cancer at the same site.
  - 138 were referred:
    - 51 (31%) with benign findings did not require biopsy.
    - Of 87 biopsies, 49 (30%) were malignant and 38 (23%), benign.
- Referral to Oncology for MDT discussion was advised in 8 (2%).
- The email alert system failed to alert breast radiology on 8 occasions (2%).

**Conclusion**:  The automated referral pathway BREAST1 resulted in a 51% reduction of referrals to the Breast Unit with resultant benefit to patients and clinicians. For those referred, there were high PPVs for malignancy (29.6% for referral, 56% for biopsy) with timely assessment and diagnosis.

**References**

1. Incidental Breast Lesions Detected on Computed Thorax Tomography—Necdet Poyraz Ganime Dilek Emlik, Suat Keskin, Havva Kalkan; J Breast Health 2015; 11: 163–7. 10.5152/tjbh.2015.2656
2. The incidence and outcome of incidental breast lesions detected by computed tomography A Hussian A Gordon-Dixon H Almusawy, P Sinha A Desai; Ann R Coll Surg Engl 2010; 92: 124–126. 10.1308/003588410X12518836439083

## 3B.1 Can a specificity-focused PERFORMS case set affect the performance of breast screening readers?

### Yan Chen^1^; Jonathan J James^2^; Eleni Michalopoulou^1^; Iain T Darker^1^

#### ^1^University of Nottingham; ^2^Nottingham University Hospitals NHS Trust

##### ***Correspondence***: Yan Chen

***Breast Cancer Research*** 2023, **25 (Suppl 2)**:3B.1

**Background:** All breast screening readers in the UK are required to participate in an external quality assurance scheme using test sets, known as PERFORMS. Recent work has shown that PERFORMS accurately reflects real-life mammography reading performance, indicating that it can be a useful tool in maintaining the high reading standards of the NHS breast screening programme 1–2. Each test set consists of sixty cases that are typically enriched with challenging cancers as well as normal and benign studies and are made available twice per year.

**Methods:** In October 2021, an innovative change was made to the scheme with a case set designed to test and assist the readers in identifying mammographic features that do not need recall for further investigation, aimed at tackling a growing number of false positive recalls. This novel optional 'specificity set' was delivered in the same way as a traditional PERFORMS set.

**Results:** 409 readers across the UK examined the cases. 317 of them took part in pre- (SA15 Part1) and post- (SA16 Part1) specificity rounds. The results showed that the recall rate was significantly lower in SA16 Part1 (34.5%) than in SA15 Part1 (37.1%) amongst those who undertook the specificity set, while the correct return to screen rate was significantly higher in SA16 Part1 (88.0%) than in SA15 Part1 (83.3%) amongst those who undertook the specificity set.

**Conclusion:** The specificity set was shown to be a very useful exercise, highlighting that an individual’s performance can be altered by implementing targeted training programmes.

**References**

1. Chen Y, James JJ, Michalopoulou E, Darker TI, Jenkins J. The relationship between missed breast cancers on mammography in a test-set based assessment scheme and real-life performance in a National Breast Screening Programme. Eur J Radiol. 2021 Sept; 142:109881. 10.1016/j.ejrad.2021.109881
2. Chen Y, James JJ, Cornford E, Jenkins J. The Relationship between Mammography Readers’ Real-Life Performance and Performance in a Test Set–based Assessment Scheme in a National Breast Screening Program. Radiol Imaging Cancer. 2020 Sep 25;2(5):e200016. 10.1148/rycan.2020200016.

## 3B.2 A comparison of contrast enhanced mammography and magnetic resonance imaging in the evaluation of breast disease: A systematic review

### Bethan Williams

#### Anuerin Bevan University Health Board

##### ***Correspondence***: Bethan Williams

***Breast Cancer Research*** 2023, **25 (Suppl 2)**:3B.2

**Design**: Systematic review of quantitative studies with a narrative synthesis.

**Background**: Accurate assessment and timely diagnosis of breast cancer is crucial for successful treatment and good prognosis (1,2).

Previous research has shown promise of CEM being comparable to MRI as a problem-solving tool for breast disease (3). Limited systematic reviews appraising this subject. Review aims to collate English language literature regarding comparison of CEM and MRI at evaluating breast disease, determining the modalities comparability in breast disease evaluation.

**Methodology**: A systematic review of quantitative studies was undertaken. CINHAL, Embase, Medline, Cochrane Library, Joanna Briggs Institute, Web of Science, TRIP, Prospero and Scopus. Two reviewers independently assessed studies for methodological quality.

**Results**: Nine moderate to high quality prospective and retrospective comparative studies, with a total of 853 participants and 1514 lesions were located. No studies which addressed overall diagnostic accuracy or sizing accuracy noted any statistically significant difference (p-value =  > 0.05) between CEM and MRI. Varying results were noted with regards to sensitivity and specificity, some studies showed statistically significance whereas others demonstrated no difference.

**Conclusion**: There is consistent evidence to suggest that CEM is comparable to MRI in the evaluation of breast disease. Research suggests MRI is slightly more sensitive, however, CEM is more specific. Sizing lesion accuracy was considered comparable, although both modalities overestimated the size of lesions when compared with histopathology. The use of CEM in multi-focal breast cancer has not been addressed in this review, however, for lesion classification and preoperative staging CEM is considered suitably comparable to MRI.

**References**

1. Drukteinis JS, et al. Beyond Mammography: New Frontiers in Breast Cancer Screening. Am J Med. 2013;126(6):472–79
2. Loberg M. et al. Benefits and harms of mammography screening. Breast Ca Res. 2015;17(1)
3. Patel BK, et al. Contrast Enhanced Spectral Mammography: A Review. Semm in Ultrasound, CT and MRI: 2018;39(1);70–9

## 3B.3 Temporal enhancement features on contrasted enhanced mammography (CEM): Comparison with breast MRI

### Malavika Rajeev; Sarah Savaridas Awarded best short paper

#### University of Dundee

##### ***Correspondence***: Malavika Rajeev

***Breast Cancer Research*** 2023, **25 (Suppl 2)**:3B.3

**Introduction**: Contrast enhanced mammography (CEM) is a functional imaging technique with similar accuracy to MRI. (1,2) Time-intensity curves derived from MRI images provide additional functional information. (3) We sought to produce similar data for CEM and compare with respective MRI curves.

**Methods:** This retrospective image-analysis study included women with enhancing mass-lesions on CEM and comparative MRI studies. Early MLO and delayed MLO views were acquired, 3 and 9 min post-contrast administration respectively. CEM lesions were segmented using freehand and ellipsoid-ROIs on initial and delayed MLO views by a radiologist blinded to MRI. The mean, 90th and 99th centile greyscale values (GSV) were recorded, and temporal change calculated. Differences between the CEM temporal enhancement according to MRI-curve type were calculated using a Mann Whitney U test.

**Results:** 55 lesions were identified, 19 produced type-1 curves, one a type-2 curve and 35 type-3 curves. The solitary type-2 curve lesion was excluded from statistical analysis. Lesions with MRI type-1 curves demonstrated increasing CEM 90th and 99th centile GSVs, lesions with type 3 curves demonstrated decreasing CEM 90th and 99th centile GSVs. Whilst mean values demonstrated less variation between cohorts, all values differed significantly between cohorts, *p* < 0.05. Differences in CEM temporal enhancement was greatest for Ellipsoid-ROI 99th centile: temporal GSV 4.58 vs − 9.97, *p* = 0.001 and Ellipsoid-ROI 90th centile: temporal GSV 2.58 vs − 7.91, *p* = 0.002, for type-1 and type-3 curves respectively.

**Discussion:** Significant differences in CEM temporal GSV are demonstrated between MRI curve types. Results were most promising for ellipsoid-ROIs and 99th and 90th centile GSVs.

**References**

1. Daniaux M, de Zordo T, Santner W, Amort B, Koppelstätter F, Jaschke W, et al. Dual-energy contrast-enhanced spectral mammography (CESM). Vol. 292, Archives of Gynecology and Obstetrics. Springer Verlag; 2015. p. 739–47.
2. Xiang W, Rao H, Zhou L. A meta-analysis of contrast-enhanced spectral mammography versus MRI in the diagnosis of breast cancer. Thorac Cancer. 2020 Jun 1;11(6):1423–32.
3. Wang LC, DeMartini WB, Partridge SC, Peacock S, Lehman CD. MRI-detected suspicious breast lesions: Predictive values of kinetic features measured by computer-aided evaluation. American Journal of Roentgenology. 2009 Sep;193(3):826–31.

## 3B.4 Evaluation of an AI tool to measure mammographic density for use in a future FAST MRI trial

### Sandra Gomes^1^; Mark Halling-Brown^1^; Ken Young^1^; Matthew Trumble^1^; Lyn Jones^2^; Katherine Klimczak^2^; Jan Rose^3^; Tony Timlin^2^; Lucy Warren^1^

#### ^1^Royal Surrey NHSFoundation Trust; ^2^North Bristol NHS Trust; ^3^Independent Cancer Patients' Voice

##### ***Correspondence***: Sandra Gomes

***Breast Cancer Research*** 2023, **25 (Suppl 2)**:3B.4

**Background:** Supplemental screening trials of FAST MRI will require validated assessment of mammographic-density to identify inclusion criteria. A research AI tool has previously been developed to predict breast density from processed mammograms (1). The AI tool requires validating prior to consideration for use in future studies.

**Aim:** To evaluate the accuracy and reliability of a previously developed research AI tool, for use in a future FAST MRI trial.

**Methods:** Processed mammograms acquired on Hologic X-ray systems and their Volpara (version 1.5.4) 5th Edition BI-RADS density classifications from unprocessed images were collected for 12,627 women from two UK screening sites(2). Inclusion criteria specified mammograms from women most likely to benefit from supplemental screening (age 50–55, first screen). BI-RADS classifications from the AI tool were compared, to those from Volpara (ground truth). 95% confidence intervals, calculated using 1000 bootstrap samples were used to compare performance across age groups and ethnicity.

**Results:** The percentage of women in BI-RADs categories a, b, c and d respectively correctly categorised by the AI tool was 85% (95%CI: 82–88), 87% (95%CI: 86–88), 81% (95%CI: 80–82) and 83% (95%CI: 82–85).

Within each BIRADs category, the 95% confidence intervals across age and ethnicity overlapped, indicating the difference in performance was not statistically significant.

The distribution of cases was 6%, 42%, 34% and 18% for BIRADs a, b, c and d for Volpara. This compared with 9%, 42%, 32% and 17% for the AI tool.

**Conclusions:** These results demonstrate comparable performance of the AI tool at mammographic-density categorisation to the ground truth Volpara.

**References**

1. Lucy M. Warren, Peter Harris, Sandra Gomes, Matthew Trumble, Mark D. Halling-Brown, David R. Dance, Louise Wilkinson, Ros Given-Wilson, Kenneth C. Young, "Deep learning to calculate breast density from processed mammography images," Proc. SPIE 11513, 15th International Workshop on Breast Imaging (IWBI2020), 115131C (22 May 2020); 10.1117/12.2561278
2. Mark D. Halling-Brown, Lucy M. Warren, Dominic Ward, Emma Lewis, Alistair Mackenzie, Matthew G. Wallis, Louise S. Wilkinson, Rosalind M. Given-Wilson, Rita McAvinchey, and Kenneth C. Young, "OPTIMAM Mammography Image Database: A Large-Scale Resource of Mammography Images and Clinical Data," Radiology: Artificial Intelligence 2021 3:1; https://pubs.rsna.org/doi/full/10.1148/ryai.2020200103

## 3B.5 Imaging and breast cancer characteristics influence mammography artificial intelligence output and detection

### Sarah Lewis; Ziba Gandomkar; Phuong Dung Yun Trieu; Seyedamir Tavakoli-Taba; Melissa Barron; Zhengqiang Jiang

#### The University of Sydney, Faculty of Medicine and Health

##### ***Correspondence***: Sarah Lewis

***Breast Cancer Research*** 2023, **25 (Suppl 2)**:3B.5

**Purpose:** The value of Artificial Intelligence (AI) models for breast cancer detection are being robustly debated in professional discourse yet the quality of image input, segmentation and demographic data is often overlooked or underreported alongside performance results. This study investigates a range of image and cancer characteristics that affect the performance of mammography-based AI models (Globally-aware Multiple Instance Classifier (GMIC); Global–Local Activation Maps (GLAM)).

**Methods:** An Australian data set of 856 screening mammography cases with a biopsy-proven malignancy were viewed by two expert breast radiologists who segmented the cancer location, aided by the pathological report. From this data, concordance values were computed and calculated using Lin’s concordance correlation coefficient and were assessed against projection type (Medio-lateral oblique (MLO), Crandio-caudal (CC)), breast density (BD) and cancer size. The annotations and concordance levels were then matched to the saliency maps of GLAM and GMIC.

**Results:** Chi-squared analysis shows concordance values between radiologists were higher for the CC view compared with MLO (P = 0.0001), and higher concordance for lower BD cases (P = 0.0016). ANOVA analysis shows significantly greater concordance for larger cancers (P = 0.03). The two AI models performed strongest when ‘almost perfect’ concordance was used, as opposed to weaker levels of concordance measured by saliency map overlap between radiologists and AIs.

**Conclusion:** The evaluation of the performance of AI models should include a multi-factorial approach to understanding the quality of input information (segmentation, concordance, views, density and cancer sizes). Segmentation and concordance can assist in demonstrating the AI output fluctuations and trustworthiness.

## 3B.6 A comparison of digital mammography and phase contrast tomography for visualisation of breast cancers

### Sarah Lewis^1^; Sharon Alexander^1^; Patrick Brennan^1^; Timur Gureyev^2^; Mary Rickard^1^; Darren Lockie^3^; Jane Fox^4^; Seyedamir Tavakoli-Taba^1^

#### ^1^The University of Sydney; ^2^The University of Melbourne; ^3^BreastScreen Victoria; ^4^Monash Health

##### ***Correspondence***: Sarah Lewis

***Breast Cancer Research*** 2023, **25 (Suppl 2)**:3B.6

**Purpose**: Two-dimensional (2D) digital mammography (DM) is the current gold standard in breast cancer screening and diagnostic imaging however limitations exist with superimposition tissues and poor contrast between healthy and cancerous tissues, particularly in dense breasts. Phase-contrast computed tomography (PCT) is a novel three-dimensional (3D) imaging approach utilising both absorption properties and refraction information of X-rays. PCT has the potential to provide additional information for breast cancer diagnosis^1^. This study aims to assess the clinical usefulness of PCT imaging compared with DM via radiological subjective visualisation of excised cancers.

**Methods**: Thirty patients underwent mastectomies, and the excised samples were subsequently imaged with PCT. The pre-surgery DM images were obtained as well as the post-surgical histological results. The 30 cases included benign cases, 22 breast cancers of varying types and a range of breast densities. A training package to allow radiologists to interpret the PTC images was developed and after completion, 2 expert breast imaging radiologists read the DM and PCT images, giving a rating of confidence as to visualisation of the cancers.

**Results**: The results show that most of the lesions were adequately visualised and detectable in PCT and DM (88%, 19/22 cases). Lower confidence was expressed with visualising high-grade DCIS lesions and LCIS lesions in DM imaging, compared with PCT and lower confidence of visualisation of Invasive Lobular Carcinoma with PCT than DM. A pictorial essay of cases is included.

**Conclusion**: PCT subjective visualisation of excised cancers was equal to DM, with new research underway to image women pre-surgery.

**Reference**

- ^1^Gureyev, TE, Nesterets, YI, Baran, PM, Taba, ST, Mayo, SC, Thompson, D, et al. "Propagation-based x-ray phase-contrast tomography of mastectomy samples using synchrotron radiation". *Med Phys*.**46**(12):5478–87. 2019. [10.1002/mp.13842]

## P01 Development of phantoms for protocol optimisation and quality assurance testing for a multi-centre abbreviated magnetic resonance imaging breast study

### Sian Curtis^1^; Holly Elbert^1^; Jonathon Delve^1^; Sam Stewart-Maggs^1^; Anna Wang^2^; Liz O'Flynn^3^; Sadie McKeown-Keegan^4^; Maria Schmidt^5^; Mark Halling-Brown^6^; Lyn Jones^4^

#### ^1^University Hospitals Bristol and Weston NHS Foundation Trust; ^2^University of Oxford; ^3^St George’s University Hospitals Foundation Trust; ^4^North Bristol NHS Trust; ^5^Royal Marsden NHS Foundation Trust and Institute of Cancer Research; ^6^Royal Surrey County Hospital NHS Foundation Trust

##### ***Correspondence***: Sian Curtis

***Breast Cancer Research*** 2023, **25 (Suppl 2)**:P01

**Background:** Previous studies have indicated that aggressive cancers, not well visualised on mammograms, can be identified on an abbreviated magnetic resonance imaging (MRI) breast protocol called FAST MRI [1,2].

The aim of this study was to design and develop dedicated phantoms for protocol optimisation and standardised quality assurance testing for a future multi-centre FAST MRI study.

**Fig. 1:**
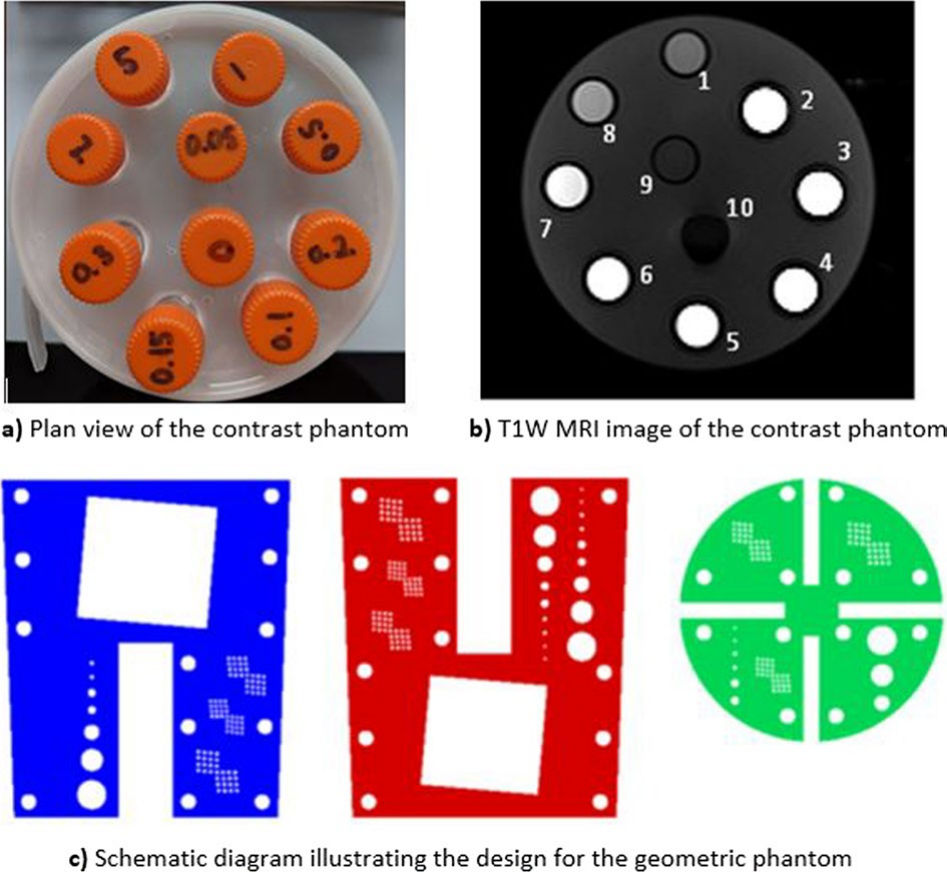
.

**Methods**: A contrast phantom was developed by testing the responsiveness of MRI contrast agents to small changes in clinical sequence parameters and assessing their stability and reproducibility (Fig. 1a and b).

A geometric phantom was developed by investigating different construction methods and target designs with comparison to expected clinical scan parameters (Fig. 1c).

**Fig. 2:**
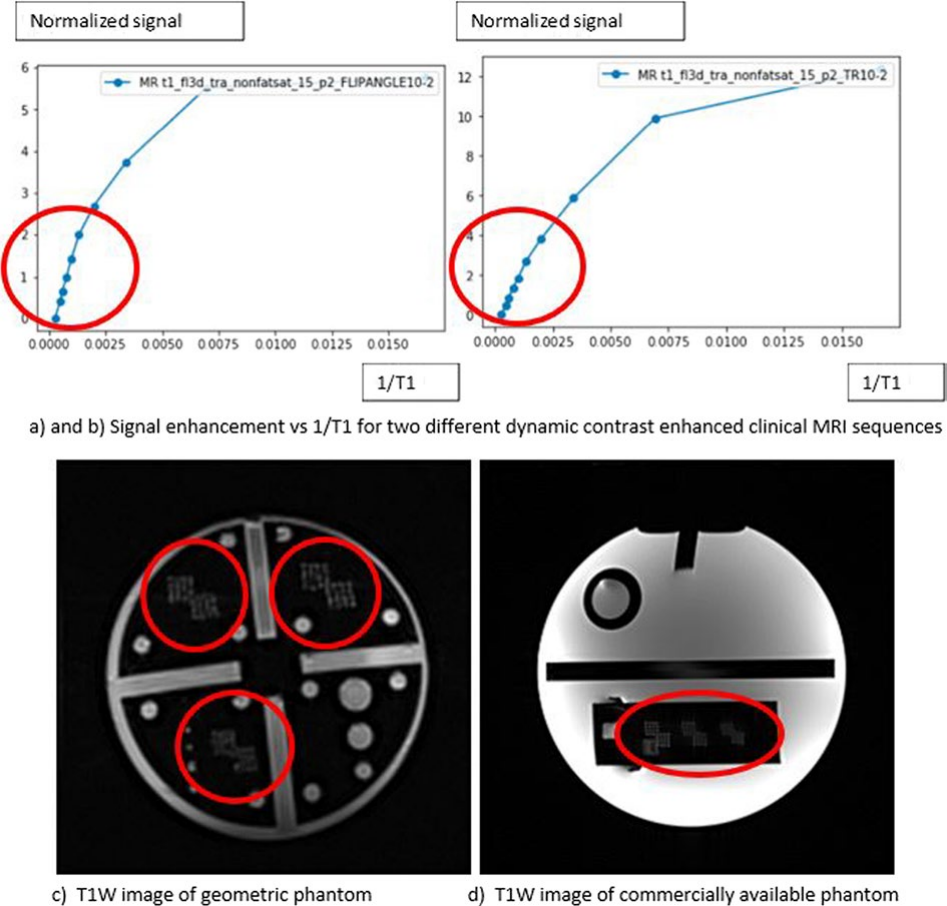
.

**Results:** Contrast phantom: The measured T1 values agreed with the literature for Gadolinium and Nickel Chloride solutions and were found to be independent of temperature and position within the magnetic field. Signal enhancement showed strong sensitivity to changes in clinical sequence parameters (Fig. 2a and b) and a linear relationship existed between signal enhancement and concentration for T1 values in the range 25–3000 ms.

Geometry Phantom: A design was chosen which included a range of resolution test targets to allow swift visual evaluation and in-depth analysis and performance tracking (Fig. 2c and d).

**Conclusion:** A contrast and geometry phantom were designed and assessed for optimisation of FAST MRI breast sequences and quality assurance testing; both phantoms fit inside a standard breast MRI coil. The phantoms have the potential to be incorporated into NHSBSP technical guidance for MRI equipment quality assurance testing.

**References**

1. Geach R, Jones LI, Harding SA, Marshall A, Taylor-Phllips S, McKeown-Keegan S, Dunn JA. The potential utility of abbreviated breast MRI (FAST MRI) as a tool for breast cancer screening: a systematic review and meta-analysis. Clinical Radiology. 2021; 76(2): 154.e11–154.e22
2. Vinnicombe S, Harvey H, Healy NA, Papalouka V, Schiller A, Moyle P, Kilburn-Toppin F, Allajbeu I, Sharma N, Maxwell AJ, Payne N, Graves M, Gilbert FJ. Introduction of an abbreviated breast MRI service in the UK as part of the BRAID trial: practicalities, challenges, and future directions. Clinical Radiology. 2021; 76(6): 427–433

## P02 Learning to read FAST MRI: Qualitative interviews with groups experienced reading mammograms

### Rebecca Geach; Lyn Jones; Sam Harding

#### North Bristol NHS Trust

##### ***Correspondence***: Rebecca Geach

***Breast Cancer Research*** 2023, **25 (Suppl 2)**:P02

**Purpose:** Abbreviated breast MRI (abMRI) is being introduced in breast screening trials and clinical practice, particularly for women with dense breasts. Upscaling abMRI provision requires the workforce of mammogram readers to be able both learn and subsequently implement the reading of the abMRI images. The present study explores the acceptability of the implementation of developed reader training, and the barriers and facilitators to participating in the training programme and subsequently reading the study training images in a work/NHS context familiar to the individual participants.

**Methods:** Fourteen mammogram readers participated in semi-structured interviews. Template analysis using the a priori implementation framework, COM-B was undertaken.

**Results:** The training day was well received. Participants identified that their varying ranges of knowledge and experience (capability) was accounted for, whilst feeling included. Participation in the research was appreciated by all, but especially those new to reading MRI.

Radiographers commented that learning to read and understand the FAST MRI images was motivational, and this helped drive implementation. It was noted that organisational leadership is needed to fully enable change in practice. COVID-19 was commented on in relation to its impact on image reading.

**Conclusions:** The project demonstrates that production of training for reading abMRI images and subsequent implementation of changes to practice needs to be carefully planned. Changes must be led by the needs of staff undertaking the tasks. When this is achieved the engagement in training is positive and the barriers are more readily removed or mitigated for both individuals and organisations.

## P03 Take a break: Should we be implementing breaks in DBT reporting sessions?

### Adnan Taib^1^; George Partridge^1^; Michael Michell^2^; Yan Chen^1^

#### ^1^University of Nottingham; ^2^King's College Hospital NHS Foundation Trust

##### ***Correspondence***: Adnan Taib

***Breast Cancer Research*** 2023, **25 (Suppl 2)**:P03

**Purpose:** We compared changes in diagnostic accuracy and fatigue levels over a Digital Breast Tomosynthesis (DBT) reporting session, with and without breaks.

**Materials:** 45 National Health Service breast screeners, from 6 breast screening centres, who participated in the UK PROSPECTS trial (NCT03733106) from December 2020 to April 2022, read a malignant-loaded set of 40 DBT cases while eye tracked in this prospective cohort study: 21 screeners had a break in their reporting session, 24 screeners did not. The eye tracker recorded eye-blink duration, assessed as an objective fatigue measure. Subjective fatigue questionnaires were completed before and after the session (unit: %). Changes in diagnostic accuracy, objective and subjective fatigue measures overtime and between cohorts were analysed (α-level was set at 0.05).

**Results:** Participant screeners had a median of 10 years post-training breast screening experience and the mean time to report all 40 cases was 105.8 min. Screeners in the no break cohort reported greater levels of mean subjective fatigue (no break: 44% vs. break: 33%; p = 0.04) which was related to a greater average blink duration (no break: 296 ms vs. break: 286 ms; p < 0.001). Blink duration also increased as the trial progressed for the no break cohort only (p < 0.001). No evidence of a difference was identified in diagnostic performance between the groups (p = 0.92).

**Conclusions:** Implementing a break during a 2-h DBT reporting session resulted in lower levels of subjective and objective fatigue. Breaks did not impact diagnostic accuracy; but this may be related to the extensive experience of the screeners.

## P04 Developing QA and QC protocols for 3D automated breast ultrasound (ABUS)

### Clara Márquez Moreno^1^; Nicholas Payne^2^; Oliver Morrish^1^; Fiona Gilbert^2^

#### ^1^Cambridge University Hospitals NHS Foundation Trust; ^2^University of Cambridge

##### ***Correspondence***: Clara Márquez Moreno

***Breast Cancer Research*** 2023, **25 (Suppl 2)**:P04

**Introduction**: The Breast Screening—Risk Adaptive Imaging for Density (BRAID) trial is a multicentre study focused on women within the NHS breast screening programme (NHSBSP) who have radiographically dense breast tissue. BRAID is investigating the potential benefit of supplemental imaging techniques to improve cancer detection. One of the modalities used in BRAID is 3D Automated Breast Ultrasound (ABUS).

**Method**: Ultrasound systems used clinically within the NHSBSP are tested following NHSBSP Publication No 70 however, this publication only covers quality assurance of 2D ultrasound systems. Therefore, we are developing a testing procedure for physicists and users using 3D ABUS systems, building on the guidance provided by national standards [1,2] and advice from the manufacturer of the equipment [3].

**Results**: The three Invenia ABUS 2.0 scanners (GE Healthcare) used within the BRAID trial are checked by physics on a six-monthly basis while user testing is carried out on a weekly and monthly basis. We are collecting, analysing, and assessing the utility of the data obtained and the tests performed during these visits. An outline of the current protocol and example results from this testing will be presented.

**Conclusions**: Initial physics and users written procedures are working documents. They are reviewed and updated based on physics and local data and feedback obtained from clinical users. The aim is that, by the end of the trial, this work will produce quality assurance and quality control protocols that could be adopted nationally to test ABUS systems.

**References**

1. Dall B, Dudley N, Hanson M, Moore S, Seddon D, Thompson W, Prashant V. Guidance Notes for the Acquisition and testing of Ultrasound Scanners for Use in the NHS Breast Screening Programme (NHSBSP Publication No 70). NHS Cancer Screening Programmes 2011.
2. Dudley N, Evans T, Hoskins P, Watson A, Starritt H. Quality Assurance of Ultrasound Imaging Systems (IPEM Report No 102). Institute of Physics and Engineering in Medicine 2010.
3. Model UC-551 m Instruction Manual, ATS Laboratories, A CIRS Company Norfolk, VA).

## P05 Enabling AI research, development and validation using the OPTIMAM mammography imaging database (OMI-DB)

### Dominic Ward; Lucy Warren; Zahida Zahoor; Andrew Joiner; Alistair Mackenzie; Emma Lewis; Ken Young; Mark Halling-Brown

#### Royal Surrey NHS Foundation Trust

##### ***Correspondence***: Lucy Warren

***Breast Cancer Research*** 2023, **25 (Suppl 2)**:P05

**Background:** The development and validation of Artificial Intelligence (AI) to improve the outcomes of breast screening depends on the availability of well-curated, representative databases (1). OMI-DB (2) was created to provide an annotated dataset to facilitate R&D. Screening mammograms and associated clinical data were collected for screen-detected cancers, interval cancers and large samples of routine-recall cases from UK screening centres since 2011. The collection is continuous.

**Results/Usage:** OMI-DB comprises 6,441,765 mammograms from 426,629 cases; a breakdown of screening cases is presented in Table 1.

**Table 1:** Counts of screening data available in OMI-DB (correct as of 10/01/2023)

Case classification	Number of cases	Number of collection sites	Number of studies	Number of images
Malignant	17,986	8	71,898	396,532
Benign	9658	8	38,856	255,898
Prior to interval cancer	3104	6	6104	31,984
Normal	377,897	8	1,009,856	5,480,533

OMI-DB is used in virtual clinical trials investigating the effect of factors on breast cancer detection and evaluating cancer characteristics and breast density. More recently, several studies have evaluated algorithms at different stages of development, from prototypes to commercial products. Unseen validation subsets have been retained for independent model validation. OMI-DB and its associated infrastructure are currently supporting multiple AI trials, evaluations and deployments (e.g. the AIMS project; (3)). OMI-DB has been shared with 70 groups, primarily for the development of machine learning.

**Discussion**: A national-scale research database has been developed. The provision of processed/unprocessed images, its large size, the availability of NBSS data and expert-determined ground truth are essential for the safe development and validation of AI.

The availability of sequential-screening events and interval cancers presents many research opportunities, including whether an abnormality could have been detected earlier. The database is continuously expanding to ensure data are representative of screening centres across the NHSBSP.

**References**

1. Debelee TG, Schwenker F, Ibenthal A, Yohannes D. Survey of deep learning in breast cancer image analysis. Evol Syst. 2020 Mar; 11:143–163. Available from: 10.1007/s12530-019-09297-2
2. Halling-Brown MD, Warren LM, Ward D, Lewis E, Mackenzie A, Wallis MG, Wilkinson LS, Given-Wilson RM, McAvinchey R, Young KC. OPTIMAM mammography image database: a large-scale resource of mammography images and clinical data. Radiol Artif Intell. 2020 Nov 25;3(1):e200103. Available from: 10.1148/ryai.2020200103
3. Imperial College London. AI in Mammography Screening (AIMS) [internet]. 2023 [cited 2023 Jan 10]. Available from: https://www.imperial.ac.uk/global-health-innovation/what-we-do/research/data-science-and-analytics/artificial-intelligence/ai-in-mammography-screening-aims/

## P06 Diagnostic performance of synthetic mammogram alone or with digital breast tomosynthesis Vs full-field digital mammogram in breast cancer screening: A systematic review

### Wasim Hamad^1^; Jonathan Myles^1^; Michael J Michell^2^; Fiona Gilbert^3^; John Loveland^4^; Stephen W Duffy^1^

#### ^1^Queen Mary University of London; ^2^King’s College Hospital; ^3^University of Cambridge; ^4^Royal Surrey County Hospital

##### ***Correspondence***: Wasim Hamad

***Breast Cancer Research*** 2023, **25 (Suppl 2)**:P06

**Background:** Digital Breast Tomosynthesis (DBT) has been shown to be effective in breast cancer screening. However, the use of DBT and full field digital mammography (FFDM) together exposes the screenee to increased radiation. A synthetic mammogram (S2D) is a two-dimensional image that can be constructed from DBT. In this review, we evaluated the performance of S2D alone or in conjunction with DBT compared to FFDM alone or with DBT.

**Methods:** A systematic search was conducted on Embase and Medline up to January 2023. Screening was conducted by two reviewers, with disagreement resolved by consensus. Studies were included if they included only screening participants and reported on the performance of S2D either alone or in combination with DBT. Studies were excluded if they included symptomatic patients, imaging was diagnostic, or if they included patients with a history of breast cancer.

**Results:** We identified 3241 records, of which 93 underwent full-text screening, and 16 were finally included. In studies reporting comparisons of cancer detection rates (CDR) between DBT + S2D and FFDM, CDRs ranged from 5.9 to 13.5/1000 for DBT + S2D. The range for FFDM was 3.5 to 9.1/1000. In studies reporting comparisons of sensitivity, the sensitivities for DBT + S2D were 69–94%, and those for FFDM 45–92%. Specificities ranged from 68 to 98% for DBT + S2D, and from 60 to 98% for FFDM. Comparison of DBT + S2D with DBT + FFDM and comparison of S2D with FFDM were consistent with these.

**Conclusion:** S2D showed similar or better performance when used with DBT compared to FFDM in breast cancer screening.

## P07 Sub-lesion level temporal enhancement characteristics of lesions on CEM: A comparison with MRI

### Calum Gray^1^; Tom MacGillivray^1^; Sarah Savaridas^2^

#### ^1^University of Edinburgh; ^2^University of Dundee

##### ***Correspondence***: Sarah Savaridas

***Breast Cancer Research*** 2023, **25 (Suppl 2)**:P07

**Introduction**: Contrast enhanced mammography (CEM) is a functional imaging technique with similar accuracy to MRI (1). However, additional functional data can be derived from MRI images using time-intensity curves, and due to the heterogeneity of breast cancers, analysis is performed at sub-lesion level to identify the most aggressive features. It may be possible to assess temporal enhancement on CEM. Initial publications compared the enhancement level of initial view (invariably CC) with subsequent view (invariably MLO). (2, 3) Results were promising but limited by the compound effect caused by assessing the greyscale levels of three-dimensional lesions using differing two-dimensional views. Subsequent work has successfully quantified change in enhancement using an early and delayed MLO views. (4) However, to date all research has been limited to assessing the change in enhancement of an entire lesion.

**Rationale:** In a proof-of-principle study of 15 cases, we demonstrated that it was possible to achieve registration with good agreement between early and delayed MLO views. This study will progress that work and perform assessment of temporal enhancement of CEM images at a sub-lesion level and compare this to respective MRI curves.

**Methods:** CEM images were performed as part of prospective imaging studies, all patients had contemporaneous MRIs. MLO views were acquired 3 and 9 min after contrast injection. Rigid and non-rigid registration techniques will be applied to spatially align the images. Subtraction images with colour maps will be produced. Foci demonstrating the most aggressive temporal enhancement features will be segmented. CEM and MRI temporal enhancement features will be compared.

**References**

1. Xiang W, Rao H, Zhou L. A meta‐analysis of contrast‐enhanced spectral mammography versus MRI in the diagnosis of breast cancer. Thoracic Cancer. 2020;11(6):1423–32.
2. Deng C-Y, Juan Y-H, Cheung Y-C, Lin Y-C, Lo Y-F, Lin G, et al. Quantitative analysis of enhanced malignant and benign lesions on contrast-enhanced spectral mammography. The British Journal of Radiology. 2018:20170605.
3. Liu Y, Zhao S, Huang J, Zhang X, Qin Y, Zhong H, et al. Quantitative Analysis of Enhancement Intensity and Patterns on Contrast-enhanced Spectral Mammography. Scientific Reports. 2020;10(1).
4. Xu W, Zheng B, Chen W, Wen C, Zeng H, He Z, et al. Can the delayed phase of quantitative contrast-enhanced mammography improve the diagnostic performance on breast masses? Quant Imaging Med Surg. 2021;11(8):3684–97.

## P08 FAST MRI interpretation-training: Using formative assessment to map the learning curve of multi-professional NHS breast screening programme (NHSBSP) mammogram-readers and to optimise diagnostic accuracy for FAST MRI (grant-funded by the National Breast Imaging Academy)

### Lyn Jones^1^; Andrea Marshall^2^; Rebecca Geach^1^; Tony Timlin^1^; Elizabeth O'Flynn^3^; Sadie McKeown-Keegan^1^; Sarah Vinnicombe^4^; Sian Taylor-Phillips^5^; Mark Halling-Brown^6^; Janet Dunn^2^

#### ^1^North Bristol NHS Trust; ^2^Warwick Clinical Trials Unit; ^3^St George's University Hospitals NHS Foundation Trust; ^4^Gloucestershire Hospitals NHS Foundation Trust; ^5^University of Warwick; ^6^Royal Surrey NHS Foundation Trust

##### ***Correspondence***: Lyn Jones

***Breast Cancer Research*** 2023, **25 (Suppl 2)**:P08

**Background**: A FAST MRI interpretation-training programme was adapted for remote e-learning delivery and used to train multi-professional NHSBSP mammogram-readers. The training (current iteration) included formative assessment through interpretation of an enriched dataset (ground-truth feedback provided immediately following participants’ reading of each scan).

**Methods**: Per-breast analysis was obtained overall and for each reader. Differences in accuracy, sensitivity and specificity across reader groups were analysed using a multilevel generalised mixed model to account for multiple readers per case and restricted cubic splines (4 knots to the number of cases read) to plot participants’ learning curves.

**Results**: 43 NHSBSP mammogram-readers completed the training. 22 interpret breast MRI in their clinical role (Group 1) and 21 do not (Group 2). 7/22 in Group 1 had previously undertaken FAST MRI training as a research participant and 13/22 had not, whilst 7/21 in Group 2 had previously undertaken FAST MRI training and 14/21 had not.

Overall sensitivity was 83% (95%CI 81–84%; 1994/2408), specificity 94% (95%CI 93–94%; 7806/8338) and readers’ agreement with the true outcome kappa = 0.75 (95%CI 0.74–0.77).

Group 1 readers showed similar sensitivity (84%) to Group 2 (82% p = 0.14), but higher specificity (94% v. 93%, p = 0.001).

**Table:** 

	Total	Group 1	Group 2
Measure			
Concordance (Accuracy)	9800/10746 (91%)	5065/5498 (92%)	4735/5248 (90%)
True positive rate (Sensitivity)	1994/2408 (83%)	1034/1232 (84%)	960/1176 (82%)
True negative rate (Specificity)	7806/8338 (94%)	4031/4266 (94%)	3775/4072 (93%)

Specificity (p < 0.001) and accuracy (p = 0.003) improved during formative assessment for all groups of readers, but sensitivity did not (p = 0.24).

**Conclusions**: Two days’ remote e-learning enabled multi-professional mammogram-readers, including those new to FAST MRI interpretation (Group 2), to achieve sensitivity and specificity within benchmarks published for full-protocol breast MRI (1).

**Reference**

1. Sickles E, D’Orsi C. ACR BI‐RADSâ Follow‐up and Outcome Monitoring. In ACR BI-RADSâ Atlas, Breast Imaging Reporting and Data System. 5th ed. Reston, VA: American College of Radiology; 2013. Available from: https://www.acr.org/Clinical‐Resources/Reporting‐and‐Data‐Systems/Bi‐Rads/Permissions

## P09 How long does it take to read a mammogram?

### George Partridge^1^; Iain Darker^1^; Jonathan James^2^; Michael Michell^3^; Yan Chen^1^

#### ^1^University of Nottingham; ^2^Nottingham Breast Institute, Nottingham University Hospitals (NUH); ^3^Kings College Hospital

##### ***Correspondence***: George Partridge

***Breast Cancer Research*** 2023, **25 (Suppl 2)**:P09

**Background**: Research shows that use of digital breast tomosynthesis (DBT) can improve diagnostic accuracy compared to digital mammography (2DDM) alone, however, DBT images are more complex and the reporting time is longer.

**Purpose**: To investigate differences in screening reporting time using 2DDM compared to DBT + 2DDM and DBT + S2D (synthetic 2D), and the effect of screener experience level by employing a large-scale dataset. Additionally, to investigate how DBT reporting time changes over a three-year period as the readers' screening experience with DBT increases.

**Methods:** PROSPECTS is a national UK prospective trial (NCT03733106) investigating the efficacy and cost-effectiveness of DBT. From January 2019-November 2022, 63 NHS breast screeners from 6 participating breast screening centres double read these clinics, equating to 41,324 2DDM and 12,084 DBT + 2D images, and recorded their reporting session durations. T-tests, ANOVAs and regression are employed to determine statistical significance, as appropriate (α-level was set at 0.05).

**Results**: All data has been collected and results show that the mean time to report a DBT case is double that of a 2DDM case (2.78 min vs. 1.27 min, respectively; p = 0.002). The time to report DBT + S2D cases was statistically equivalent to DBT + 2DDM cases (2.88 min vs. 2.68 min, respectively; p = 0.08). The experience data will be included in the conference presentation.

**Conclusions**: Analysis of this large-scale dataset shows that DBT reporting time is greater than 2DDM alone and that DBT + S2D and DBT + 2DDM reporting time is equivalent. We hypothesise that the reporting time will reduce with accrued experience using DBT as a screening tool.

## P10 Radiologists performance in DBT using online and PACS-based education tools for breast cancer detection

### Sarah Lewis; Melissa Barron; Phuong Dung Yun Trieu

#### The University of Sydney, Faculty of Medicine and Health

##### ***Correspondence***: Sarah Lewis

***Breast Cancer Research*** 2023, **25 (Suppl 2)**:P10

**Background:** Digital Breast Tomosynthesis (DBT) is a mainstream imaging method for early breast cancer detection and ongoing education is vital for strong diagnostic performance. This study investigated the diagnostic performance of readers interpreting a DBT test set in two modes: through PACS (Picture Archiving and Communication System) or online directly through the BREAST (Breast Screen Reader Assessment Strategy) cloud-based platform.

**Methods:** A DBT test set consisting of 10 biopsy-proven cancer and 20 normal case was read by Group 1: 30 readers (23 radiologists, 7 trainees) using PACS and entering their results on the BREAST platform; and Group 2: 23 readers (17 radiologists, 6 trainees) readers without PACS and direct annotation onto BREAST. Readers marked any suspicious lesions on the best corresponding DBT slice and rated the lesions for levels of malignancy suspicion. Readers’ performances were compared with truth data and evaluated in specificity, sensitivity, ROC AUC and JAFROC FOM. Both had the average experience of 5 years reading DBT cases.

**Results:** There was no significant difference between two reading modes between Group 1 and 2 for any metric (P > 0.05). Radiologists performed slightly better without PACS in all metrics, with a significant result in ROC AUC (0.79-vs-0.74; P = 0.027).

**Conclusion:** Readers demonstrated equivalent performance when reading DBT cases via PACS and without PACS, with an improved ROC AUC performance for radiologists. Online education platforms such as BREAST that have evolved past PACS technology can be very effective for training and self-assessment and can provide greater access for learning.

## P11 Self-assessment modules in mammography, lessons learned and future innovations

### Patrick Brennan^1^; Moe Suleiman^2^; Ziba Gandomkar^2^; Mary Rickard^2^; Noelle Clerkin^3^

#### ^1^DetectedX & University of Sydney; ^2^DetectedX; ^3^Belfast Breast Unit

##### ***Correspondence***: Patrick Brennan

***Breast Cancer Research*** 2023, **25 (Suppl 2)**:P11

**Background:** Self-assessment modules (SAMs), have educated expert breast radiologists for three decades. Following millions of user test set interactions we can now document what SAMs have achieved and outline where future innovations should be focussed.

**Methods:** BREAST and DetectedX test sets which provide a variety of mammographic SAMs have been used by clinicians in Australasia, Asia, Europe and the US as a method to improve early detection of breast cancer whilst cultivating abilities to recognise normality. Whilst it is generally agreed that SAMs have provided an essential clinical service, it is also recognised that evolution is required to embrace modern day innovations such as artificial intelligence (AI).

**Results:** Clinical performance significantly improves with the use of SAMs regardless of experience and training of the user, with particular benefits shown for cancer detection. In addition the multiple interactions of clinicians with SAMs has yielded valuable insights [1,2] resulting in more than 100 publications and multiple research student theses. New knowledge on radiologist behaviours and characteristics that affect image interpretation has been provided. However, it is now timely for SAMs to reassess their place in the modern educational arena in terms of access, duration, accreditation, interactivity and AI. The data presented summarises achievements and describes potential future pathways.

**Conclusions:** Much has been achieved with SAMs, however with the emergence of new technologies and user expectations they must now evolve and embrace new opportunities that will help optimise and tailor educational deliveries.

## P12 Breast screening for transgender people: How confident are we? Awarded Caroline Roney Prize

### Emily Gilbert

#### Nottingham Breast Institute

##### ***Correspondence***: Emily Gilbert

***Breast Cancer Research*** 2023, **25 (Suppl 2)**:P12

**Purpose:** Many transgender people are invited for breast screening in the UK (1), yet there is limited research on how we can support this community. NHS England provides guidance on service adjustments to accommodate transgender individuals (2), but there is currently no specific training advised for mammographers. This study aims to explore how confident mammographers are in screening transgender people. This includes confidence in their knowledge, using appropriate terminology, and understanding how the service could adapt. The aim is to also explore whether mammographers have had training around this topic, and if they feel they require any.

**Methods:** A quasi-structured questionnaire was carried out by a volunteer sample of mammographers (N = 10). Some questions were presented as a Likert scale (3), and were analysed as ordinal data using a measure of central tendency (mean). Other questions were open, and were analysed thematically.

**Results:** Mammographers had low confidence levels in using appropriate terminology (mean = 4.4, whereby 0 = not confident at all and 10 = very confident). Mammographers also had fairly low confidence in understanding why transgender people may or may not be invited for screening (mean = 5.6). Emerging themes included feeling anxious about getting things wrong, and feeling unsure about adaptations. None of the mammographers had received training around the topic, and 80% said they would like training.

**Conclusions:** Improvements should be made in teaching mammographers about the transgender community in relation to breast screening. Increasing mammographers' knowledge and confidence could lead to service improvements for transgender people. The author has produced a pilot information leaflet for existing mammographers and trainees.

**References**

1. Public Health England. Breast screening: Guidance for breast screening mammographers [Internet]. gov.uk. [cited 2022 Dec 22]. Available from: https://www.gov.uk/government/publications/breast-screening-quality-assurance-for-mammography-and-radiography
2. NHS England. NHS Population Screening: Information for trans and non-binary people [Internet]. gov.uk. [cited 2022 Dec 22]. Available from: https://www.gov.uk/government/publications/nhs-population-screening-information-for-transgender-people/nhs-population-screening-information-for-trans-people
3. McLeod SA. Likert scale [Internet]. Simply Psychology. [cited 2022 Dec 22]. Available from: https://www.simplypsychology.org/likert-scale.html

## P13 Can we de-escalate surgical treatment for DCIS?

### Aisling Eves^1^; Andrew Pieri^2^; Ross McLean^2^; Nerys Forester^2^

#### ^1^Newcastle University; ^2^Royal Victoria Infirmary

##### ***Correspondence***: Aisling Eves

***Breast Cancer Research*** 2023, **25 (Suppl 2)**:P13

**Background:** DCIS accounts for 20% of malignancies diagnosed by the breast screening programme and is primarily managed by surgical excision. This study aims to investigate how often DCIS is fully removed via core biopsy, thereby negating the need for surgery*.*

**Methods:** This was a single-centre retrospective cohort study of 101 consecutive breast screened patients diagnosed with DCIS who underwent surgical excision. All patients diagnosed with DCIS had radiological abnormalities < 15 mm. Clinical, radiological, and histological data were collected from patients who had been diagnosed within a 5-year period, and a complete excision by core biopsy was defined as 0 mm of DCIS found in the surgical specimen.

**Results:** Complete DCIS excision following core biopsy was 21.8% (n = 22). The median mammographic size of DCIS was 8 mm (range: 4–14 mm), median number of cores was 8 (3–16) and median biopsy weight was 1.82 g (1.1–7.5 g). There were no significant differences in mammographic size (10 mm, p = 0.06), number of cores (9, p = 0.14), or biopsy weight (2.73, p = 0.26) for those who had incomplete excision. Complete excision was seen in 40% of low-grade, 29% of intermediate-grade, and 16% of high-grade DCIS cases (p = 0.19).

**Conclusion:** There are no clear factors which predict complete excision by core biopsy in screen-detected DCIS. It is possible that DCIS < 15 mm could be excised with VAE techniques but further investigations are needed to determine this. In low-grade DCIS further work could be considered due to higher rates of complete excision with core-biopsy. We would recommend following relevant guidelines to proceed to surgical excision where appropriate.

## P14 Clinical outcomes for patients diagnosed with DCIS through the breast screening programme

### Aisling Eves^1^; Andrew Pieri^2^; Ross McLean^2^; Nerys Forester^2^

#### ^1^Newcastle University; ^2^Royal Victoria Infirmary

##### ***Correspondence***: Aisling Eves

***Breast Cancer Research*** 2023, **25 (Suppl 2)**:P14

**Background:** This study investigated the clinical outcomes (re-excision rates, radiotherapy usage, and presence of invasive cancer) for patients following surgical excision of small-volume DCIS.

**Methods:** This was a single-centre retrospective cohort study of 101 consecutive breast-screened patients diagnosed with DCIS who underwent surgical excision. All DCIS patients had radiological abnormalities < 15 mm. Clinical, radiological, and histological data were collected from patients diagnosed within 5-years, and ASCO guidelines for margin involvement < 2 mm was used to guide the need for re-excision.

**Results:** Breast conservation surgery was performed in 94.1% (n = 95). Following surgical excision, 74 (73.27%) patients had complete DCIS excision (> 2 mm margin), 4 (4.0%) had margins 1–2 mm, and 17 (16.84%) had margins < 1 mm. The median size of DCIS in the specimen sample was 4 mm. In 86% of patients with involved margins (n = 18), the mammogram underestimated DCIS size by a median of 12.5 mm (range: 1–42 mm). Of the patients with involved margins, 11 (10.9%) had a re-excision, with 6 (50%) requiring two re-excisions to achieve complete DCIS excision. Post-operative radiotherapy was provided to 53 (52.48%). Four (3.97%) patients had invasive ductal carcinoma on surgical excision which was not present on core biopsy. Recurrence of DCIS in the same site occurred in 1 patient (1%), 1-year after first DCIS diagnosis.

**Conclusion:** Breast conservation surgery is safe in DCIS patients, with low rates of re-excision, recurrence and upstaging to invasive cancer. Furthermore, the median size of DCIS found in the specimens of patients with total DCIS removal was small suggesting that VAE could be used to totally remove DCIS in these patients.

## P16 Radiography advanced practitioners' clinical background: Impact upon interpretation performance

### Noelle Clerkin^1^; Patrick Brennan^2^; Chantal Ski^1^; Ruth Strudwick^1^

#### ^1^University of Suffolk; ^2^University of Sydney

##### ***Correspondence***: Noelle Clerkin

***Breast Cancer Research*** 2023, **25 (Suppl 2)**:P16

**Purpose:** The efficacy of a breast cancer screening programme relies on efficient analysis of mammograms. Readers that interpret mammograms must meet the high standards of their peers (1,2). This study will for the first time identify the clinical background implications that impact the performance of UK Radiography Advanced Practitioners (RAP). This data will assist in identifying norms for the assessment of diagnostic efficacy.

**Methods**: The performance of 22 UK-based RAPs reading a cloud-based test set of 60 mammographic cases with known truth was assessed using the software platform DetectedX. Sensitivity and specificity values were established for each RAP, T or Mann–Whitney tests were used to explore the impact of clinical background on image interpretation accuracy.

**Results:** Reader sensitivity was significantly affected by the weekly volume of reads undertaken (P ≤ 0.0001), number of annual readings (P ≤ 0.0001), average reading session length (P ≤ 0.0001), type of cancer presented on imaging (P ≤ 0.0001), breast speciality (P ≤ 0.0001) and years in the role (P ≤ 0.0001). Specialists in breast imaging (P ≤ 0.04) and their preferred time of day to read (P ≤ 0.04) impacted their specificity. Normative performance values were indicated.

**Conclusions:** For the first time this work has identified specific features that impact the performance of UK RAPs interpreting mammograms. Through identified causal agents for varying performance suggested are ways in which future diagnostic activity can be optimised. These findings provide a framework for the assessment of standards, available to all RAPs.

**References**

1. Kundel, H., Nodine, C. and Carmody, D., 1978. Visual Scanning, Pattern Recognition and Decision-making in Pulmonary Nodule Detection. *Investigative Radiology*, 13(3), pp.175–181.
2. Rawashdeh MA, Lee WB, Bourne RM, Ryan EA, Pietrzyk MW, Reed WM, et al. Markers of good performance in mammography depend on number of annual readings. Radiology. 2013;269(1):61–7

## P17 Experience as an international mammographer working in the UK- comparing practice between Nigeria and UK

### Chizoba Rita Okeke; Ginikanwa Njoku

#### Royal Shrewsbury and Telford Hospital NHS Trust

##### ***Correspondence***: Ginikanwa Njoku

***Breast Cancer Research*** 2023, **25 (Suppl 2)**:P17

Breast cancer affects women of all races without exception even though severity and survival rate are often diverse. In Nigeria about two thirds of women with breast cancer are diagnosed at an advanced stage, with the possibility of metastatic spread (Akaro-Anthony et al., 2010).

A mammographer performs breast imaging techniques that produce mammographic radiographs for diagnosis (American Society of Radiologic Technologist, 2017).

In Nigeria, the breast screening programme is performed by radiographers with the additional mammogram-specific training which is comparable to what is found in the United Kingdom; however, the UK screening programme also makes use of trained assistant practitioners which is not obtainable in Nigeria (Lawal et al., 2015).

The breast screening programme in Nigeria invites women between the ages of 40 to 70 years, and this is justified by the fear that in Nigeria, a higher percentage of breast cancer cases are seen in younger age groups than in developed world ((Jedy-Agba et al., 2012). The mode of invitation is through public awareness campaigns, but majority of the women in the population do not frequently participate in mammography screening due to high cost and religious belief. The screening programme in Nigeria encourages women to get screened every two years (Lawal et al., 2012).

However, the UK breast screening programme advice women to have breast screening mammogram, once every 3 years and is currently inviting women between the ages of 50 and 70 years for breast screening.

**References**

1. Akarolo-Anthony SN, Ogundiran TO, Adebamowo CA (2010) Emerging breast cancer epidemic: evidence from Africa. Breast Cancer Res. 12 Suppl 4:S8.
2. American Society of Radiologic Technologists (ASRT). The Practice Standards for Medical Imaging and Radiation Therapy US: American Society of Radiologic Technologists; 2017:1–29.
3. Jedy-Agba, E. et al*.* (2012) "Cancer incidence in Nigeria: A report from population-based cancer registries," *Cancer Epidemiology*, 36(5).
4. Lawal, O., Murphy, F.J., Hogg, P., Irurhe, N. and Nightingale, J. (2015). Mammography screening in Nigeria—A critical comparison to other countries. *Radiography*, 21(4), pp.348–351. 10.1016/j.radi.2015.03.015.
5. Maroni, R., Massat, N.J., Parmar, D., Dibden, A., Cuzick, J., Sasieni, P.D. and Duffy, S.W. (2020). A case–control study to evaluate the impact of the breast screening programme on mortality in England. *British Journal of Cancer*. 10.1038/s41416-020-01163-2.
6. Pittathankal, A. and Davidson, T. (2010). Care pathways for patients with breast cancer. *Trends in Urology, Gynaecology & Sexual Health*, 15(2), pp. 10–13. 10.1002/tre.144.

## P18 Women's perspectives of molecular breast imaging

### Helen Elliott^1^; Alison Bray^1^; Nerys Forester^1^; Will Jones^2^; Clare Lendrem^2^; Timothy Powell^1^; Jason Scott^3^

#### ^1^Newcastle Upon Tyne Hospitals NHS Foundation Trust, University of Newcastle; ^2^NIHR Newcastle In Vitro Diagnostics Co-operative; ^3^Northumbria University

##### ***Correspondence***: Helen Elliott

***Breast Cancer Research*** 2023, **25 (Suppl 2)**:P18

**Background**: Mammography used for breast screening has poor sensitivity in dense tissue ^(1)^. Retrospective studies suggest that Molecular Breast Imaging (MBI), used for screening in the USA but not the UK, has superior diagnostic accuracy ^(2,3)^. Patient perspectives of MBI are unknown and crucial to understand feasibility of adoption into the NHS.

**Method:** Semi-structured interviews with screened and unscreened women exploring the acceptability of MBI. Data were analysed thematically.

**Results:** Five themes were generated from 19 interviews: (1) scan duration, (2) radiation dose, (3) equity of access, (4) comfort in familiarity and (5) need for shared decisions relating to risk. Participants found the 40-min scan duration to be acceptable. Radiation dose was also acceptable, especially once participants understood that higher breast density was linked to cancer risk. Some participants were concerned about access issues such as parking if MBI scans were hospital-based, raising issues around equitable access. Participants expressed obtaining comfort in existing screening processes with which they were familiar, and participants with experience of nuclear medicine tests were less concerned about radiation dose. Finally, participants placed considerable trust in the NHS to evaluate tests, pointing to a need for uncertainty in screening decisions to be more effectively discussed to support shared decision making.

**Conclusion:** MBI is an acceptable breast imaging modality for UK women. Women wish to be offered personalised, risk-based screening, with tests that offer favourable risk–benefit ratios. High-quality patient information enabling informed decision-making is essential. Further work is needed to understand how MBI will fit into existing screening pathways.

**References**

1. Kolb TM, Lichy J, Newhouse JH. Comparison of the Performance of Screening Mammography, Physical Examination, and Breast US and Evaluation of Factors that Influence Them: An Analysis of 27,825 Patient Evaluations. Radiology. 2002;225(1):165–75.
2. Kim BS, Moon BI, Cha ES. A comparative study of breast-specific gamma imaging with the conventional imaging modality in breast cancer patients with dense breasts. Ann Nucl Med. 2012;26(10):823–9.
3. Rhodes DJ, Hruska CB, Conners AL, Tortorelli CL, Maxwell RW, Jones KN, et al. Journal club: molecular breast imaging at reduced radiation dose for supplemental screening in mammographically dense breasts. AJR Am J Roentgenol. 2015;204(2):241–51.

## P20 Evaluating the radiological features and characteristics of interval breast cancers in a UK breast screening programme

### Cheryl Stubbs^1^; Amy Symons^2^; Elizabeth Muscat^1^; Elizabeth O' Flynn^1^; Cathie Morris^1^

#### ^1^St George's Healthcare NHS Trust; ^2^Kingston University

##### ***Correspondence***: Cheryl Stubbs

***Breast Cancer Research*** 2023, **25 (Suppl 2)**:P20

**Background**: Every year approximately 6000 women have an interval cancer diagnosed 1. 80% of interval cancers have no sign of the subsequent cancer on the screening mammogram, however 20% do, representing a false negative interval cancer (FNIC). We have evaluated the radiological features and characteristics of FNIC’s in an NHSBSP breast unit.

**Methods**: A retrospective service evaluation of all FNIC was performed between 1/1/2016 and 1/1/2022. For each FNIC anatomical position, breast density, mammographic appearance and image quality were evaluated.

**Results**: 33% of FNIC underwent paired arbitration: 87% were not recalled for assessment and 13% were which were subsequently discharged to routine screening. 3% had clinical symptoms.

**Anatomical position:** 64% the abnormality was seen on both views in the upper outer quadrant, 17.5% in the lower inner quadrant, 16% posteriorly, 10% retroareolar and 6% at the site of post-surgical change.

**Breast density:** 14% (BI-RADS A), 46% (BI-RADS B), 36% (BI-RADS C) and 4% (BI-RADS D).

**Mammographic appearance:** 61% asymmetry: 28.5% slowly developing asymmetry, 74% focal asymmetry, 26% only seen on one view, 6% associated with calcification. Figure 2 demonstrates the distribution of tumour characteristics within each breast density.

**Image quality:** 11.5% were impacted by image quality at the site of the abnormality.

**Conclusions**: Review of screen detected cancers may yield additional cases which can facilitate further learning. Training in recognition of significant clinical symptoms and a review of the clinical symptoms protocol could prevent further cases.

**Reference**

1. Public Health England (2021) NHS screening: helping you decide: London: Public Health England

## P21 When clips migrate following a vacuum-assisted procedure, does the technique affect the distance of migration?

### Hannah Giles; Andrea Da Silva; Geetanjali Kakar; Neil Upadhyay

#### Imperial College Healthcare NHS Trust

##### ***Correspondence***: Hannah Giles

***Breast Cancer Research*** 2023, **25 (Suppl 2)**:P21

**Learning objective:** Assess the difference in the distance of clip migration between lateral arm biopsy approach (LABA) and vertical conventional biopsy approach (CBA) when performing vacuum assisted procedures.

**Background:** Vacuum-assisted procedures under stereotactic or tomosynthesis guidance allows sampling of mammographically detected abnormalities.

Clip migration occurs in up to 44% of cases [1]. There are several potential contributary factors, such as the accordion effect. It is hypothesised that approach, LABA or CBA, may affect migration.

The poster aims to compare the mean distance of clip migration in CBA and LABA procedures using the T-test, to ascertain whether the degree of clip migration is affected.

**Results:** The Radiology Information System was searched with key term “migr” to account for “migration” or “migrated”, yielding 70 results between 2015 and 2022.

55 were CBA, 11 were LABA and 4 were excluded.

Breast density (BIRADS classification), age, approach and clip migration distance were recorded. The mean distance of migration for CBA was 28 mm and 24 mm for LABA (p = 0.49).

**Conclusion:** There was no statistically significant difference in the mean distance of migration between CBA and LBA approaches, suggesting that technique does not affect the extent of clip migration.

**Limitations:** The search used the term “migr” and therefore reports using alternative vocabulary to describe migration would not have been captured. As this is likely to affect cases of CBA and LABA equally, it would be unlikely to introduce bias.

**Reference**

1. Weaver et al. Insights into Imaging (2021) 12:193 10.1186/s13244-021-01136-w

## P22 What lies outside the breast- incidental extra-mammary findings on MRI

### Lauragh Mc Carthy; Elisabetta Giannotti

#### Addenbrookes Hospital, Cambridge

##### ***Correspondence***: Lauragh Mc Carthy

***Breast Cancer Research*** 2023, **25 (Suppl 2)**:P22

**Background:** Breast MRI has superior anatomical resolution and larger field of view than other breast imaging techniques and is the only one to include extramammary structures.

According to previous studies 16.8%–34% of breast MRI examinations demonstrate incidental extramammary findings. Benign lesions are the most common findings. Malignant lesions need to be excluded especially as many breast MRI patients have newly diagnosed breast cancer and such significant findings would influence patient management.

The aim of this study was to determine the prevalence and site of extramammary findings on breast MRI.

**Method:** Retrospective review of all Breast MRI’s over a 12 month period from January to December 2022 in a single institution was performed.

929 examinations were reviewed.

**Results:** Extramammary incidental findings were identified in 98 of the 929 examinations (10.5%).

6.1% were malignant findings (3 lung, 2 bone, 1 chest wall); one was a new diagnosis of sarcoidosis (1/98 1%) and one a deep venous thrombosis (1/98 1%).

The most common incidental finding was hepatic cysts 61.2% (60/98). The next most common findings were retrosternal goitre, hepatic haemangioma and cardiovascular findings each identified in 6/98 6.1%.

**Conclusion:** When reporting breast MRI it is essential to examine and review extra-mammary structures to exclude the presence of findings which might have a significant clinical impact on patient management and care.

**References**

- Niell BL, Bennett D, Sharma A, Gazelle GS. Extramammary findings on breast MR examinations: frequency, clinical relevance, and patient outcomes. Radiology 2015;276(1):56–64
- Rinaldi P, Costantini M, Belli P, et al. Extra-mammary findings in breast MRI. Eur Radiol 2011;21(11):2268–2276.

## P24 Contrast enhanced mammography guided biopsy (CEMGB) to the rescue: A case-based step by step guide to this versatile technique for biopsy of lesions occult on standard imaging ***Awarded Best Poster***

### Rachel Sun; Ramona Menezes

#### Nottingham University Hospitals

##### ***Correspondence***: Rachel Sun

***Breast Cancer Research*** 2023, **25 (Suppl 2)**:P24

**Background:** Contrast Enhanced Mammography (CEM) is a dual energy acquisition technique using a single compression. It relies on tumour neo-angiogenesis, highlighted by intravenous iodinated contrast. A low energy exposure is performed followed by a high energy exposure (above the k-edge of iodine) using copper filtration. These images are processed to generate a recombined image that supresses background tissue and highlights areas of tumour enhancement.

Our unit introduced CEM in 2013 and recently implemented CEM guided biopsy—CEMGB. CEMGB utilises CEM principles alongside conventional stereotactic guidance. It allows targeting of lesions that are occult on conventional imaging but visible on recombined images.

The procedure is performed using standard stereotactic techniques that most radiographers will be familiar with. The process is straight-forward when staff have been trained appropriately and are familiar with CEM. It can be performed in breast clinic with minimal preparation, and the potential for diagnosis at first patient attendance.

**Objectives:** To present a CEM case and provide a step-by-step guide to performing a CEMGB with the General Electric (GE) Pristina mammography machine. We provide tips and tricks and potential pitfalls.

**Conclusion:** CEMGB is a promising technique to biopsy lesions occult on conventional imaging. It has the potential to be a cheaper, faster and more widely available alternative to MRI guided breast biopsy.

## P25 Percutaneous vacuum-assisted excision (VAE) of breast lesions of uncertain malignant potential (B3) as an alternative to diagnostic surgical excision. Experience of a large screening service

### Maria Carrillo; Heba Hussein; Marianna Telesca

#### Worcestershire Acute Hospitals

##### ***Correspondence***: Maria Carrillo

***Breast Cancer Research*** 2023, **25 (Suppl 2)**:P25

**Objective:** Standard treatment for B3 lesions has been surgical excision. The benign post-surgical outcome of majority of B3 lesions called for a less invasive approach such as VAE. This retrospective study aims to assess the impact of VAE as alternative to diagnostic surgical excision in the management of screen detected B3 lesions in a large screening service (population 150,000).

**Method:** 82 B3 lesions on 14 g core biopsy were referred for VAE between 2018 and 2021. Pathological information on initial 14 g biopsy and final VAE/surgical histology were obtained. VAE data collection included: image guidance (mammography/ultrasound), needle size (7/10 g), number and weight of samples.

**Results:** On VAEs 33/82 lesions had no atypia and 49/82 had atypia. VAE was performed under mammography (48/82) or ultrasound (34/82). 44 VAEs performed with 10 g needle with an average of 11.8 samples (mean sample weight 3.75 gr). 38 VAEs performed with a 7 g needle with an average of 11.4 samples (mean sample weight 5.12 gr).

On final histology, 11/82 cases (13.4%) were upgraded to malignancy requiring surgical management. 61/82 (74.4%) cases did not require further treatment and returned to routine screening. 10/82 (12.1%) cases were offered increased annual surveillance for 5 years as per NHSBSP guidelines. Up to the time of the abstract, no new malignancy has occurred at site of VAE.

**Conclusion:** 71/82 (86.5%) cases had VAE as final treatment and did not require any further surgical management demonstrating VAE is an effective method to treat screen detected B3 breast lesions.

**Reference**

- Elisabetta Giannotti, Jonathan J. James, Yan Chen, Rachel Sun, Amanjot Karuppiah, Julia Yemm, Andrew H. S. Lee. Effectiveness of percutaneous vacuum-assisted excision (VAE) of breast lesions of uncertain malignant potential (B3 lesions) as an alternative to open surgical biopsy. European Radiology. 2021 Dec;31(12):9540–9547.

## P27 Is the 3 mm tolerance for axillary node biopsy still adequate for indication of axillary disease? Retrospective audit

### Linda Deane; Lucy Cielecki; Sue Williams; Marie Metelko; Mahmoud Alkouly; Umit Aksoy; Jayne Vaughan; Sian Burley; Elizabeth Barlow; Ellen Cobby

#### Shrewsbury and Telford Hospital NHS Trust

##### ***Correspondence***: Linda Deane

***Breast Cancer Research*** 2023, **25 (Suppl 2)**:P27

**Background:** A 3 mm tolerance for cortical thickness on axillary lymph nodes is a standard measurement used as one of the thresholds to decide if potentially suspicious for disease. Our department conducted an audit of the ultrasound outcomes for lymph node involvement in the axilla after several unexpected positive post-surgical cases with previously negative axillae on ultrasound, were obtained. This could impact the patient directly if further axillary surgery was required.

**Method:** 12 months of ultrasound results were compared to the pathology results for surgical axillary biopsy, lymph node sampling and surgical axillary clearance.

**Results:** Forty-seven cases out of 388 cases were false negative. Sensitivity was 41.25% and specificity was 91.56%.

**Analysis:** Nineteen cases were excluded due to morphological data unavailable at the time of the audit. Twenty-eight cases analysed either revealed disease too small to be visualised, positive nodes not in the axilla, not biopsied by a second consultant when returning for biopsy procedure and learning difficulties. All these cases were conducted by different consultants on different ultrasound units. No identifiable trend was seen.

**Conclusion:** The sensitivity is in keeping with peer review investigations and therefore the 3 mm threshold for identifying possible disease is still adequate. Any learning points from individual cases were taken forward to aid with service improvement.

**References**

1. Saffar, B., Bennett, M., Metcalf, C., & Burrows, S. Retrospective preoperative assessment of the axillary lymph nodes in patients with breast cancer and literature review. *Clinical Radiology. 2015. 70*(9), 954–959. 10.1016/j.crad.2015.04.019
2. Bedi, D. G., Krishnamurthy, R., Krishnamurthy, S., Edeiken, B. S., Le-Petross, H., Fornage, B. D., Bassett, R. L., & Hunt, K. K. Cortical Morphologic Features of Axillary Lymph Nodes as a Predictor of Metastasis in Breast Cancer: In Vitro Sonographic Study. *American Journal of Roentgenology. 2008. 191*(3), 646–652. 10.2214/AJR.07.2460
3. Luparia, A., Campanino, P., Cotti, R., Lucarelli, D., Durando, M., Mariscotti, G., & Gandini, G. Role of axillary ultrasound in the preoperative diagnosis of lymph node metastases in patients affected by breast carcinoma. *Radiologia Medica. 2010. 115*(2), 225–237. 10.1007/s11547-009-0465-8
4. Elmore, L. C., Appleton, C. M., Zhou, G., & Margenthaler, J. A. Axillary ultrasound in patients with clinically node-negative breast cancer: which features are predictive of disease? *The Journal of Surgical Research. 2013. 184*(1), 234–240. 10.1016/j.jss.2013.03.068
5. Deurloo, E.., Tanis, P.., Gilhuijs, K. G.., Muller, S.., Kröger, R., Peterse, J.., Rutgers, E. J. T., Valdés Olmos, R., & Schultze Kool, L. Reduction in the number of sentinel lymph node procedures by preoperative ultrasonography of the axilla in breast cancer. *European Journal of Cancer. 2003. 39*(8), 1068–1073. 10.1016/S0959-8049(02)00748-7
6. Sanders, M. M., Waheed, S., Joshi, S., Pogson, C., & Ebbs, S. R. The importance of pre-operative axillary ultrasound and intra-operative sentinel lymph node frozen section analysis in patients with early breast cancer—A 3-year study. *Annals of the Royal College of Surgeons of England. 2011. 93*(2), 103–105. 10.1308/003588411X12851639108196
7. Rooney, E. G., Fleming, M. M., Patel, J. G., Clifford, K., Kim, C., Chen, Z., Gillespie, T. W., Arciero, C. A., & Subhedar, P. D. Are Lymph Node Characteristics on Axillary Ultrasound Associated with ≥ 3 Positive Lymph Nodes in Patients Managed by the American College of Surgeons Oncology Group Z0011 Trial Criteria? *The American Surgeon. 2018. 84*(7), 1133–1137. 10.1177/000313481808400726

## P28 Complications of image guided breast biopsies at a breast screening hospital: A prospective review

### Sharmeen Jaffer; Janet Sedgwick; Gillian Bowman; Lucy Diss; Michael Michell; Rema Wasan; Charlotte Longman; Adam Brown; Clare Peacock; Nikhil Patel; Bhavana Batohi; Keshthra Satchithananda; Juliet Morel

#### King's College Hospital

##### ***Correspondence***: Sharmeen Jaffer

***Breast Cancer Research*** 2023, **25 (Suppl 2)**:P28

**Background:** Core biopsies are the gold standard technique for diagnostic breast biopsies in the United Kingdom.1 There are a plethora of techniques for sampling including stereotaxis, tomosynthesis, ultrasound and MRI guided vacuum assisted/core biopsies. While procedural optimisation is key, complications do arise including haematoma with/without evolution to fat necrosis, scarring or seroma(2–14.4%), infection ± abscess formation(4–6%), bruising and pain.2–5 A prospective review was performed to evaluate complication rates for x-ray guided vacuum assisted biopsies (VAB), x-ray and ultrasound guided vacuum assisted excisions(VAE).

**Methodology:** Data was collated in the form of a questionnaire for 79 patients (n = 79) who underwent a stereotactic prone or ultrasound breast VAB or VAE between February-June 2022; including bruising, haematoma and infection. Additionally a subjective pain score was recorded (1–10) immediately after the biopsy (post-biopsy) and on follow-up appointment 7–14 days post biopsy (follow-up).

**Results:** The majority of patients experienced no pain (27/79) or mild pain (24/79) with 34% and 44% recording a pain scale of 0 (post-biopsy and at follow-up respectively). On follow-up 68% (n = 52) of patients exhibited bruising and 25% (n = 19) haematoma formation. No patients presented with infection. Data for 9G VAB with 12 cores (n = 51) was further analysed, no correlation was demonstrated between pain score and haematoma formation based on breast density (A-D) and lesion location.

**Conclusion:** Post procedural haematoma formation and pain scores are higher than desirable relative to international literature.6 Implementation of post procedural compression for 10 min with the intention to reassess haematoma rates in 6 months. Infection rates are comparable to recent literature.

**References**

1. Mahoney MC, Newell MS. Jul 1 2013. Breast Intervention: How I Do It. 10.1148/radiol.13120985. RSNA. Radiology Vol. 268, No. 1
2. Chetlen AL, Kasales C, Mack J, Schetter S, Zhu J. Hematoma formation during breast core needle biopsy in women taking antithrombotic therapy. AJR Am J Roentgenol. 2013 Jul;201(1):215–22. 10.2214/AJR.12.9930. PMID: 23789678.
3. Somerville P, Seifert PJ, Destounis SV, Murphy PF, Young W. Anticoagulation and bleeding risk after core needle biopsy. AJR Am J Roentgenol. 2008 Oct;191(4):1194–7. 10.2214/AJR.07.3537. PMID: 18806164.
4. Bick U, Trimboli RM, Athanasiou A, Balleyguier C, Baltzer PAT, Bernathova M, Borbély K, et al.; European Society of Breast Imaging (EUSOBI), with language review by Europa Donna–The European Breast Cancer Coalition. Image-guided breast biopsy and localisation: recommendations for information to women and referring physicians by the European Society of Breast Imaging. Insights Imaging. 2020 Feb 5;11(1):12. 10.1186/s13244-019-0803-x. PMID: 32025985; PMCID: PMC7002629.
5. AH Lee (2021) G150. Guidelines for non-operative diagnostic procedures and reporting in breast cancer screening. Royal College of Pathologists
6. Fang M, Liu G. Feasibility and safety of image-guided vacuum-assisted breast biopsy: A PRISMA-compliant systematic review and meta-analysis of 20 000 population from 36 longitudinal studies. Int Wound J 2019; 16 (6):1506–1512

## P29 In the context of a benign or normal ultrasound biopsy result, what prompts repeat biopsy to prove benignity?

### Laura Ward; Delara Khodabakhshi; Elizabeth O'Flynn; Lisanne Khoo

#### St George's Hospital

##### ***Correspondence***: Delara Khodabakhshi

***Breast Cancer Research*** 2023, **25 (Suppl 2)**:P29

**Background:** NHS Breast Screening Programme (NHSBSP) guidance recommends repeat biopsy if a result is equivocal (1), although ‘equivocal’ is not defined. Practice regarding re-biopsy for lesions yielding B1, B1/2 and B2 (i.e. benign/normal) pathology is therefore variable.

**Objectives:** Determine the re-biopsy rate for lesions biopsied under ultrasound with a benign/normal result. Assess the patient and imaging factors influencing the decision to re-biopsy vs accepting the benign/normal result and review final pathology.

**Methods:** Ultrasound guided biopsies from a 1- year period from a single screening/symptomatic unit, yielding benign/normal outcomes, were retrospectively obtained from the Pathology database. Screening vs symptomatic, age, M/U grade, lesion size and reported needle position relative to lesion were obtained from the Picture Archiving Service, Radiology Information Service, NHSBSP database and Breast Screening clinic notes.

**Results:** 765 biopsies were benign/normal. Of these, 46 (6%) underwent a re-biopsy, with rates of 9.8% (24/245) in screening and 4.2% (22/520) in symptomatic. Likelihood of re-biopsy increased with higher M and U gradings and with lower B grading (Tables 1 & 2). 14 high M/U grade lesions did not undergo re-biopsy under ultrasound. These cases were reviewed. Patient age, lesion size and documentation of needle position did not affect decision to re-biopsy.

**Table 1:** Repeat biopsy rate, correlating M grade and initial biopsy grade

M grading	Total M grade	B1		B1/B2		B2	
	303	Total B1	Number repeated	Total B1/B2	Number repeated	Total B2	Number repeated
			(%)		(%)		(%)
M1	63	12	0 (0)	10	1 (10)	41	0 (0)
M2	121	11	2 (18.2)	21	2 (9.5)	89	0 (0)
M3	216	32	12 (37.5)	29	4 (13.8)	155	9 (5.8)
M4	5	0	0 (0)	1	1 (100)	4	0 (0)
M5	7	3	1 (33.3)	3	3 (100)	1	1 (100)

**Table 2:** Repeat biopsy rate correlating U grade and initial biopsy grade

U grading	Total U grade	B1		B1/2		B2	
	765	Total B1	Number repeated (%)	Total B1/2	Number repeated (%)	Total B2	Number repeated (%)
U2	246	15	5 (33.3)	21	2 (9.5)	209	1 (0.4)
U3	499	62	13 (21)	77	8 (10.4)	360	10 (2.8)
U4	16	4	3 (75)	2	1 (50)	10	0 (0)
U5	4	0	0 (0)	0	0 (0)	4	2 (50)
							1 formal excision
							1 resolved on repeat US

Of the 46 repeat biopsies, 6 yielded B1 (13%), 10 B1/B2 (21.7%), 28 B2 (60.9%), 1 B3 (2.2%) and 1 yielded B5b (2.2%).

**Conclusions:** This study has highlighted the importance of radiological and pathological concordance in determining whether a re-biopsy is required and the key role therefore of the MDM in reaching these decisions.

**Reference**

1. NHS BSP Publication no 49: Clinical Guidance for Breast Cancer Screening Assessment. Fourth edition, 15th November 2016

## P30 B3 lesions managed with vacuum assisted excision (VAE): Are 12 × 7G cores equivalent to 4 g of tissue?

### Rebecca Watson; Chin Lian Ng; Mags Akigbogun; Joan Butt; Vaishali Gada; Rosie Browning; Ozan Asmakutlu; Vivian Chiu; Briony Bishop

#### Buckinghamshire NHS Trust

##### ***Correspondence***: Vaishali Gada

***Breast Cancer Research*** 2023, **25 (Suppl 2)**:P30

**Purpose/Objectives:** NHS Breast Screening Programme (NHSBSP) Publication 49^1^ states that following a diagnosis of a B3 lesion vacuum assisted excision (VAE) is the procedure of choice to excise approximately 4 g (12 × 7G) of breast tissue for further evaluation. The breast imaging team wanted to assess whether 12 × 7G full notch cores yielded 4 g of tissue.

**Methods:** A prospective audit was undertaken of consecutive VAE cases, performed under ultrasound or stereotactic guidance from both NHSBSP and two week wait symptomatic sources over a 12 month period. Tissue samples retrieved were weighed and the number of core samples and needle gauge was recorded. For ultrasound guided procedures extent of lesion excision was documented. Post VAE histology was reviewed and compared to the histology yielding a B3 diagnosis.

**Results:** A total of 59 cases were recorded, 32 ultrasound guided and 27 under stereotactic guidance. There was a greater variation of cores obtained under ultrasound guidance thus a wider specimen weight range averaging 3.6 g. A minimum of 12 cores were always obtained for stereotactic guided procedures eliciting an average of 5 g.

**Conclusions:** Ultrasound guided VAE’s were performed for complete lesion excision and in some cases this was achieved in less than 12 cores, thus lowering the average specimen weight. A minimum of 4 g of tissue was consistently yielded from 12 × 7G conforming to current guidance.

**Reference**

1. Public Health England. NHS Breast Screening Programme: Clinical guidelines for breast cancer screening assessment. NHSBSP publication number 49 [Internet]. 2016 [cited 2023 Jan 10]. Available from: Clinical guidance for breast cancer screening assessment (publishing.service.gov.uk)

## P31 Evaluating the effectiveness of a breast abscess pathway at a South West London NHS tertiary centre

### Abhinav Jha; Sharon Koo; Karis McFeely; Shahrooz Mohammadi

#### St George's University Hospital, London

##### ***Correspondence***: Abhinav Jha

***Breast Cancer Research*** 2023, **25 (Suppl 2)**:P31

**Introduction**: Breast abscesses are a common breast pathology presenting to the emergency department (ED). A breast abscess pathway was implemented at this London NHS tertiary centre in September 2019 to facilitate imaging of suspected abscesses from ED. We audited referrals and patient outcomes to improve workflow and use of radiology resources.

**Method**: Retrospective audit of all breast abscess pathway referrals from September 2019 to September 2022. Radiology/clinical documentation on Powerchart and PACS was reviewed by two radiology trainees. Presenting symptoms, antibiotic prescribing, referral documentation, imaging and clinical outcomes were documented on Excel.

**Results**: 284 patients were referred, excluding 7 due to incomplete documentation.

200 (70%) patients were correctly referred with signs of abscess, 24 (8%) patients were referred with no documented clinical findings of abscess, and 60 (22%) partially fulfilled the referral criteria. 72 (36%), 0 and 8 (13%) had an abscess on imaging respectively.

Of the 25 (9%) of patients not on antibiotics at the time of imaging, 2 (10%) had abscess on imaging versus 76 (30%) of the 256 patients on antibiotics.

63 (79%) patients with abscess underwent ultrasound guided aspiration. 94 (34%) and 21(7%) had ultrasound guided and surgical procedures respectively. 4 (1%) had inflammatory cancers.

**Discussion**: 30% of referrals are inappropriately completed. Imaging findings of abscess are higher with appropriate referrals, and patients on antibiotics. Most abscesses required aspiration.

Compliance can be improved by ED teaching, and imaging request checklists. This will streamline patient care and use of radiology resources.

**Reference**

1. https://cks.nice.org.uk/topics/mastitis-breast-abscess/background-information/prevalence/

## P32 Breast ultrasound imaging in adult male patients presenting at a London NHS tertiary centre

### Abhinav Jha; Isabel Cornell; Sarah McWilliams

#### St George's University Hospital, London

##### ***Correspondence***: Abhinav Jha

***Breast Cancer Research*** 2023, **25 (Suppl 2)**:P32

Male breast masses are uncommon, with detection often surprising for patients and clinicians. The clinician’s understanding of imaging features of normal, common benign, and rarer malignant findings is crucial. All patients at our institution undergo a triple assessment with clinical history and examination and ultrasound initially. Mammography is a useful troubleshooting tool to downgrade gynaecomastia which is indeterminate on ultrasound. Core biopsies are performed on indeterminate lesions and lesions with sinister features.

We reviewed adult breast ultrasound at our institution in the year 2022. Male breast ultrasound formed a small number (4%) of all breast ultrasound conducted, with the most common indication being gynaecomastia. The majority of examinations (97%) revealed U2 findings, with the majority being gynaecomastia (65%). Other U2, U1 and U5 findings comprised 18%, 14% and 3% respectively.

We discuss tools for the recognition and differentiation on ultrasound of gynaecomastia and its nodular, dendritic and diffuse patterns; mastitis and breast abscesses; lipomas; primary breast and metastatic malignancies; axillary pathologies; fibroadenomas and pathologies more commonly encountered in female patients. We also discuss how mammography can aid in downgrading gynaecomastia with indeterminate findings to avoid unnecessary core biopsy. Finally, core biopsy should be used for indeterminate and more sinister lesions.

Whilst the majority of breast ultrasound examinations in the male breast show gynaecomastia, it is crucial to diagnose this in indeterminate cases to avoid core biopsies, with mammography a key tool for this. Furthermore, it is important to recognise other benign findings, and critically the rare malignancies.

## P33 CT detected breast lesions. What to do and how to report them

### Maria Carrillo; Farah Sadrudin

#### Worcestershire Acute Hospitals

##### ***Correspondence***: Maria Carrillo

***Breast Cancer Research*** 2023, **25 (Suppl 2)**:P33

**Objective:** In the past 10 years there has been an exponential use on CT resulting in the identification of incidental breast lesions. Due to its good contrast resolution and larger field of view than mammography breast lesions can be easily detected.

Aim: To familiarize Radiologist and Radiographers with the appearances of benign and malignant breast lesions depicted by CT.

**Method:** Through this poster we will show cases of beging, indeterminate and suspicious lesions on CT, post operative changes and differentiation with recurrence and some incidental findings on PET CT.

**Results:** After reading this poster there will be clarity on how to interpret incidental Breast lesions, how to manage them and how to report them appropriately.

**References**

- Harish M, Konda S, MacMahon H, Newstead G. Breast Lesions Incidentally Detected with CT: What the General Radiologist Needs to Know. Radiographics 2007; Octo 27 Suppl 1: S37–51
- Saeedan M, Mobara M, Arafah M,Mohammed T. Breast lesions on chest computed tomography: pictorial review with mammography and ultrasound correlation Curr Probl Diagn Radiol. 2015 Mar-Apr;44 (2):144–54.
- Georgieva M, Rennert J, Brochhausen C, Stroszczynski C, Jun E. Suspicious breast lesions incidentally detected on chest computer tomography with histopathological correlation. Breast J. 2021;27:715–722.
- Kim S, Park J. Computed tomography of the breast. Abnormal findings with mammographic and sonographic correlation. J Comput Assist Tomogr 2003 Sep-Oct;27(5):761–70.

## P34 Correlation of clinical examination, MRI, ultrasound and mammography with pathological sizes

### Mei Chan Chin^1^; Sarah Al-Ani^2^; Karen Gray^1^

#### ^1^NHS Lanarkshire; ^2^NHS Greater Glasgow and Clyde

##### ***Correspondence***: Mei Chan Chin

***Breast Cancer Research*** 2023, **25 (Suppl 2)**:P34

**Objective:** To determine how accurately the different radiological modalities and clinical examination correlated with true tumour size.

**Methods:** 96 MRI breasts with contrast performed between January 2019 and October 2020 which had preceding breast ultrasound and mammograms were obtained. PACS and Clinical Portal were used to obtain the relevant measurements.

Reported pathological sizes were presumed to be true tumour sizes. Where available, the clinical examination measurements and reported radiological measurements were obtained. We excluded any entries which did not yield malignancies with comparable measurements. We employed the Pearson correlation coefficient to determine how well clinical examination and each of the different modalities correlated with the pathological measurements.

**Results:** 41 patients with 42 tumours had reported pathological sizes post-surgical excision. There were 41 correlating available ultrasound, 39 MRI and 24 mammographic measurements. There were only 18 correlating clinical examination measurements. Average tumour size was 29.3 mm. The distribution of malignancy types were 20 lobular, 17 ductal, 4 mixed lobular and ductal and 1 mucinous in our sample. Our results were that MRI correlated the strongest with pathological size at 93%. Mammogram, ultrasound and clinical examination correlation with pathology tumour size were lower at 84%, 70% and 69% respectively.

**Conclusion:** Our sample mirrors that of published literature which indicate that MRI is more accurate than mammography or ultrasound at predicting pathologic tumour size. In our sample, ultrasound has the lowest correlation with true tumour size probably because of the relatively high number of lobular cancers which are known to be morphologically more diffuse, less-circumscribed and hence more difficult to measure.

**References**

1. Hieken TJ, Harrison J, Herreros J, et al. Correlating sonography, mammography, and pathology in the assessment of breast cancer size. Am J Surg 2001;182:351–4.
2. Lehman CD, Gatsonis C, Kuhl CK, et al. MRI evaluation of the contralateral breast in women with recently diagnosed breast cancer. N Engl J Med 2007;356:1295–303.
3. Boetes C, Mus RD, Holland R, et al. Breast tumors: comparative accuracy of MR imaging relative to mammography and US for determining extent. *Radiology*1995;**197**:743–747.
4. Leddy R, Irshad A, Metcalfe A, et al. Comparative accuracy of preoperative tumor size assessment on mammography, sonography, and MRI: Is the accuracy affected by breast density or cancer subtype? J Clin Ultrasound 2016;44:17–25.
5. Madjar H, Ladner HA, Sauerbrei W, et al. Preoperative staging of breast cancer by palpation, mammography and high-resolution ultrasound. Ultrasound Obstet Gynecol 1993;3:185–90.
6. Davis PL, Staiger MJ, Harris KB, et al. Breast cancer measurements with magnetic resonance imaging, ultrasonography, and mammography. *Breast Cancer Res Treat*1996;**37**:1–9.
7. Pritt B, Ashikaga T, Oppenhemier RG, et al. Influence of breast cancer histology on the relationship between ultrasound and pathology tumor size measurements. *Modern Pathology* 2004; 905–910

## P35 Implementation of preoperative upfront magnetic seed (magseed) localization for impalpable screen detected breast cancer

### Vrinda Sukumaran; Zoe Goldthorpe

#### Somerset NHS Foundation Trust

##### ***Correspondence***: Vrinda Sukumaran

***Breast Cancer Research*** 2023, **25 (Suppl 2)**:P35

**Background:** Magseed is an effective non-inferior alternative to wire guided localisation of impalpable breast lesions. The aim of this audit was to evaluate the effectiveness in placing Magseed at the time of biopsy in screen detected breast lesions.

**Methods:** An audit was undertaken of a prospectively maintained database of patients who attended assessment for screen detected breast lesions. Patients were selected for upfront Magseed localisation based on a protocol that defined parameters including breast density, radiological features, size and nodal status. Magseed was placed under Ultrasound guidance during their initial visit to the assessment clinic. Data was collected on biopsy findings, MDT outcome and final surgical pathology.

**Results:** A total of 20 patients were identified and audited against the criteria set out in the protocol.

All lesions in which Magseed was placed were shown to be biopsy proven malignancy. Invasive ductal carcinoma being the predominant pathology (85%). Of the 3 lesions diagnosed as lobular carcinoma no disruption to their diagnostic pathway or surgical pathology were found.

**Conclusions:** Upfront Magseed localisation is a safe, easy procedure and may improve workflow for radiology services under pressure.

**References**

1. Zacharioudakis, K. (2019, November 1). *Is the future magnetic? Magseed localisation for non palpable breast cancer. A multi-centre non randomised control study*. European Journal of Surgical Oncology. https://www.ejso.com/article/S0748-7983(19)30529-3/fulltext
2. *Overview | Magseed for locating impalpable breast cancer lesions | Advice | NICE*. (2020, November 17). https://www.nice.org.uk/advice/mib236
3. Thekkinkattil,D.(2019, September 25). A prospective,single-arm,multicentre clinical evaluation of anew localisation technique using non-radioactive magseeds for surgery of clinically occult breast lesions. 10.1016/j.crad.2019.08.018

## P36 Imaging of male breast disease—a pictorial review demonstrating a spectrum of male breast disease with radio-pathological correlation

### Joan Butt; Ozan Asmakutlu; Rosie Browning; Vivian Chiu; Vaishali Gada; Chin Lian Ng; Rebecca Watson

#### Bucks Breast Unit

##### ***Correspondence***: Vaishali Gada

***Breast Cancer Research*** 2023, **25 (Suppl 2)**:P36

The male breast can be affected by a wide range of conditions. Most cases of male patients presenting with breast lumps are due to benign causes—only 1% of cases are due to a malignant process^1^. Breast lobular development is not common in men; therefore, breast conditions related to lobular proliferation, such as fibroadenoma and invasive lobular carcinoma are extremely rare in men^2^.

Correct interpretation of imaging findings can guide clinical management by differentiating between benign and malignant processes and is critical because it alleviates patient anxiety and can avoid unnecessary procedures^2^.

The poster is a pictorial review including a variety of interesting male breast conditions which have all presented at a 2WW breast clinic within a district general hospital over a four-year period. Cases range from the benign pathologies of breast abscess and gynaecomastia to various malignant cases of invasive and non-invasive breast cancer. A very rare and unusual case of a metastatic deposit of oesophageal origin presenting as a lump within the breast will also be presented^3,4^.

A broad range of imaging modalities are included ranging from mammography and ultrasound to CT and MRI.

Clinical findings and key imaging features will be shown along with pathological correlation, demonstrating the importance of robust triple assessment in the accurate diagnosis of male breast disease.

**References**

1. Chesebro, A. Male Breast Disease: What the Radiologist needs to know. Curr Probl Diagn Radiol. 2019 Sep-Oct;48(5):482–493.Available from: 10.1067/j.cpradiol.2018.07.003. Epub 2018 Jul 29
2. Viana, M. Imaging of male breast disease: the good, the bad and the ugly – A pictorial Review. Breast Imaging. 2020 December 1; Volume 68: P45–56. Available from: 10.1016/j.clinimag.2020.06.025
3. Genç, B. Metastasis to the Male Breast from Squamous Cell Lung Carcinoma. Case Reports in Oncological Medicine Volume 2013, Article ID 593970, 4 pages. Available from: http://dx.doi.org/10.1155/2013/593970
4. Wu, S-G, Zhang. Sites of metastasis and overall survival in esophageal cancer: a population-based study. Cancer Manag Res.2017; 9: 781–788. Published online 2017 Dec 6. Available from: 10.2147/CMAR.S150350

## P37 Managing anxiety in mammography: The client and the practitioner

### Johanna Mercer^1^; Claire Mercer^2^

#### ^1^Vita Health Group: Newcastle; ^2^University of Salford

##### ***Correspondence***: Claire Mercer

***Breast Cancer Research*** 2023, **25 (Suppl 2)**:P37

**Background:** Being responsible for providing the best possible care to those attending mammography and promoting wellbeing is important (1). Anxiety plays a large role in maintaining negative well-being; an emotion individuals experience when they are worried, afraid, or tense (2).

Client anxiety contributes to factors such as pain (3–5) and reattendance rates (6–8). Educating practitioners in client wellbeing, utilising strategies to enforce this in practice, can ensure they are providing good care whilst improving wellbeing, attendance rates and a reduction in pain.

Practitioner wellbeing is equally important. Low wellbeing can lead to staff sickness (9), increasing NHS cost, and is also correlated with medical errors and decreased patient safety (10). Educating practitioners on strategies to manage anxiety, whilst adapting the workplace to help reduce anxiety, can aid in improving practitioner wellbeing and reducing sickness and errors.

**Methods:** To identify and explain the common causes of anxiety in clients and practitioners, and demonstrate knowledge to implement solutions for reducing client and practitioner anxiety.

**Results:** Incorporating relaxation techniques and procedural knowledge into client leaflets, improving practitioner communication, and adapting the mammography environment can help reduce client anxiety and increase attendance rates. For practitioners, using mindfulness and stress management techniques, communicating regularly with peers and managers, and having access to a serenity room can help reduce anxiety.

**Conclusions:** A focus on anxiety management can have positive benefits to both staff and clients within mammography services.

**References**

1. Public Health England. *Guidance for breast screening mammographers*. Available from: https://www.gov.uk/government/publications/breast-screening-quality-assurance-for-mammography-and-radiography/guidance-for-breast-screening-mammographers. [Accessed 15th August 2021].
2. Department of Health. *The relationship between wellbeing and health*. Available from: https://assets.publishing.service.gov.uk/government/uploads/system/uploads/attachment_data/file/295474/The_relationship_between_wellbeing_and_health.pdf. [Accessed 10th August 2021].
3. Brunton M, Jordan C, Campbell I. Anxiety before, during, and after participation in a population-based screening mammography programme in Waikato Province, New Zealand. NZMJ. 2005;118(1209):1–10.
4. Jackson V. Pain psychology: An overview of concepts and methods. In: Hogans BB, Barreveld AM. (eds.). *Pain care essentials*. USA: Oxford University Press; 2019. p. 75–89.
5. Maimone S, Morozov AP, Wilhelm A, Robrahn I, Whitcomb TD, Lin KY, Maxwell RW. Understanding patient anxiety and pain during initial image-guided breast biopsy. Journal of Breast Imaging. 2020 Nov;2(6):583–89.
6. Aro AR, De Koning HJ, Absetz P, Schreck M. Two distinct groups of non-attenders in an organized mammography screening program. Breast Cancer Research and Treatment. 2001 Nov;70(2):145–53.
7. Consedine NS, Magai C, Krivoshekova YS, Ryzewicz L, Neugut AI. Fear, anxiety, worry, and breast cancer screening behavior: a critical review. Cancer Epidemiology and Prevention Biomarkers. 2004 Apr 1;13(4):501–10.
8. Lagerlund M. Factors affecting attendance at population-based mammography screening. Department of Medical Epidemiology and Biostatistics; 2002.
9. Montgomery M, McCrone SH. Psychological distress associated with the diagnostic phase for suspected breast cancer: systematic review. Journal of Advanced Nursing. 2010 Nov;66(11):2372–90.
10. Hu W, Wang G, Huang D, Sui M, Xu Y. Cancer immunotherapy based on natural killer cells: current progress and new opportunities. Frontiers in Immunology. 2019 May 31;10(1):1205.

## P38 Compress with me

### Uju Olakunle

#### Doncaster and Bassetlaw Teaching Hospitals NHS Foundation Trust

##### ***Correspondence***: Uju Olakunle

***Breast Cancer Research*** 2023, **25 (Suppl 2)**:P38

**Background:** Compress with me technique has shown to be a technique that could reduce compression pain and anxiety experienced by patients during mammography examination. The discomfort of the mammographic examination can be linked to the decision to attend for screening. Therefore, interventions to reduce the discomfort caused by the mammogram are key to improve women’s adherence, whilst assuring the technical quality of the image1. This study aims to evaluate the value of targeted communication (Mammographer-patient interaction) during compression in mammography (especially in women whose breasts are particularly sensitive) and its effect in the improvement of low uptake of screening appointments.

**Method:** Data was collected from two clinical training centres between April to July 2022, using non-probability, convenience sampling method. Questionnaire was administered before the examination to 296 mammogram patients. Participants were women aged 50 to 75 years, without a history of recent breast surgical procedure or treatment, and who could perform breast self-examination. After positioning the patient, using the foot pedal, the compression was brought down until 35N, then the mammographer together with the patient manually completes the compression.

**Results:** 78% (230.88/296) of the women preferred the compress with me technique over the foot pedal controlled compression technique due to reduced pain and anxiety, 14% (41.44/296) still felt pain and discomfort but due to other various reasons, whilst 8% (23.68/296) felt indifferent about the technique.

**Conclusion:** Results suggest that under the same amount of compression force, compress with me technique gave much reduced pain and anxiety compared to foot pedal compression.

**Reference**

1. Perez-Leon D, Posso M, Louro J, Ejarque B, Arranz M, Arenas N, Maiques J, Martínez J, Maciá F, Román M, Rodríguez-Arana A, Castells X, Alcántara R. Does the patient-assisted compression mode affect the mammography quality? A within-woman randomized controlled trial. Eur Radiol. 2022 Nov; 32(11):7470–7479. 10.1007/s00330-022-08834-z. Epub 2022 May 10. PMID: 35536391

## P39 Trends in equipment faults, downtime, and cancellations in the UK breast screening programmes

### John Loveland; Alistair Mackenzie

#### Royal Surrey NHS Foundation Trust

##### ***Correspondence***: John Loveland

***Breast Cancer Research*** 2023, **25 (Suppl 2)**:P39

**Background:** To evaluate trends with time for x-ray fault rates, downtime, and equipment related cancellations in breast screening.

**Methods:** The National Coordinating Centre for Physics in Mammography (NCCPM) has created and maintained a database of mammography equipment faults for more than 30 years. Data were analysed over the period 1993 to 2021.

**Results:** The number of faults reported per x-ray set has increased from about 1.1 to a peak of 4.4 faults/unit but average downtime per fault has remained stable. Total downtime has increased from around 250 days to a peak of 1400 days. Average age of screening equipment has also increased but despite a proven link between age and fault rate this does not fully explain the change.

Digital systems have a higher fault rate than film-screen systems t(8) = 6.76, p < 0.001 and this difference is independent of equipment age. Most fault types have remained relatively constant over time such as cracked paddles but new faults such as software faults have risen rapidly over the last 15 years. This suggests that the increased number of faults are not due to better reporting rates.

**Conclusions:** From the fault database, we have been able to show trends in faults and their effect on the screening programme. It has proven to be a useful tool and has had important implications for planning in the UK breast screening programmes.

**Reference**

1. Stuffins M, Loveland J, Halling-Brown M, Mackenzie A. The relationship between age of digital mammography systems and number of reported faults and downtime. Phys Med. 2022 Jun;98:113–121. 10.1016/j.ejmp.2022.04.015. Epub 2022 May 6. PMID: 35526372.

## P40 A service evaluation of a symptomatic breast one-stop clinic

### Devakie Sookdeo

#### University College London Hospital and Kingston University

##### ***Correspondence***: Devakie Sookdeo

***Breast Cancer Research*** 2023, **25 (Suppl 2)**:P40

**Background:** 60% of women diagnosed with breast cancer present symptomatically via One-Stop Clinics (OSCs)^1^. OSCs facilitate a two-week wait referral-to-diagnosis pathway which utilises triple assessment (TA) to ensure 97% accuracy of non-operative diagnosis^2^. OSCs have been repeatedly reviewed to improve access for urgent referrals^3^.

**Aim:** Perform a service evaluation of a symptomatic breast OSC evaluating: structure, process, and output.

**Methodology:** A cross-sectional, cohort design, using quantitative, retrospective data from a convenience sample of 1157 patients attending a Central London NHS Hospital, over four-months (September-December 2019 and Tuesdays in May 2020). Permission from the Clinical Governance Committee was obtained. Data from EPIC and Carestream Vue PACS were recorded anonymously in a Microsoft Excel spreadsheet, including:

- Structure: compared three OSCs: routine (SOSC) (single-radiologist), double-OSC (DOSC) (double clinic and radiologists) and Covid-OSC (COSC) (triaged appointments) to assess appointment capacity, time-efficiency, and workflow.
- Process: assessed use of resources [digital breast tomosynthesis (DBT), needle-tests].
- Output: evaluated the impact of triaging referrals versus outcomes.

**Results:** 104 appointment weekly capacity across 77 OSCs were assessed.

- 1125(97%) patients completed TA appointments within two visits. 923(80%) received same-day diagnosis.
- 162(14%) of 992(86%) TA patients followed radiology-led discharge pathway which saved average 51-min per appointment.
- Average appointment was 140-min (8–360-min). Average waiting time for surgical consult was 39-min, and ultrasound 42–51 min.
- 52/85 DBTs downgraded mammograms. 4/85 were upgraded.
- DBT prevented 80% overdiagnosis and could save average 27–52 min per appointment.
- 95(10%) patients experienced diagnostic delays (1–55 days). 59% due to radiology time constraints, 12% patient choice.
- 557(48%) referrals required GP management, 344(30%) were non-urgent.

**Conclusion:** This unit met OSC capacity demands and diagnostic targets.

**References**

1. Ramzi, S. and Cant, P. J. (2021) ‘Comparison of the urgent referral for suspected breast cancer process with patient age and a predictive multivariable model’, *British Journal of Surgery Open,* 5(2). 10.1093/bjsopen/zraa023.
2. Batt, J., Ainsworth, R., Rayter, Z., Nickells, J. and Valencia, A. (2021) ‘Sensitivity and missed cancer rate in the symptomatic breast clinic—A retrospective cohort study of 40 323 patients’, *The Breast Journal*, 27(3), pp. 248–251. 10.1111/tbj.14134.
3. Rao, A., Razzaq, H., Panamarenko, B., Bottle, A., Majeed, A. and Gray, E. (2021) ‘Online application for self-referral of the patients with breast symptoms’, *Annals of Medicine and Surgery,* 66(2021), pp. 102372. 10.1016/j.amsu.2021.102372

## P42 Over 70 self-referral screening outcomes to Belfast breast screening unit—a retrospective study 2015- 2022

### Jenny Frazer

#### Belfast Health and Social Care Trust

##### ***Correspondence***: Jenny Frazer

***Breast Cancer Research*** 2023, **25 (Suppl 2)**:P42

**Introduction:** The Breast Screening Propgramme (BSP) invites clients up to age 70, after 70 clients can self refer. As a region Northern Ireland give cards/leaflets to clients, who attend their last invited mammogram, to promote self referral in 3 years time.

**Aim:** Examine extent ≥ 70 women self-refer in Belfast Trust during an eight-year period and study the screening outcomes in these women.

**Method:** Retrospective review of NHS Breast Cancer Screening Database (NBSS) crystal report and KC62 looking at self-referral appointments, recalls to assessment clinic and resultant cancer diagnoses in women over 70 years in Belfast Trust between 31st March 2015 and 1stApril 2022. Analysed resultant diagnosis to determine tumor grade, lymph node and hormone status on surgical specimen pathology report on Northern Ireland electronic care record (NIECR).

**Result:** Over this 8-year time period, the total number of invitations for ≥ 70 years old was 5926 of these 4544 clients attended, DNA rate of 1382 (23.3%). The clients recalled to assessment were 197 (4.3%) with a cancer detection rate of 23.3 per 1000) and Invasive cancer detection rate of 18.9 per 1000. The average whole tumor size detected was 19.64 mm with a range of 0.8–96 mm. The tumor grade is known for 75 out of 86 invasive cancers, The most common tumor grade is grade 2, most common hormone status ER positive (66/80, 82.5%)PR positive (54/80, 67.5%) Her-2 negative (70/80, 87.5%).

**Conclusion:** Approx 2.5% of screening women self-refer in the Belfast unit, proportionately higher number of over 70 women are recalled to assessment and subsequent increased cancer detection rates ≥ 70 years. Does the self referal policy of the NHSBSP cause finical and capacity burden on the screening units.

**Reference**

- Chandler, A. P., Davies, L., Gower-Thomas, K., Lewis, H., & Dillon, M. (2019). P031. Patterns of self-referral for breast cancer screening in women aged over 70 in Wales between 2005 and 2016. *European Journal of Surgical Oncology*, *45*(5), 894. 10.1016/j.ejso.2019.01.053

## P43 A retrospective study to evaluate multiple biopsies of microcalcifications identified on screening mammography

### Kerrie Power; Laura Foster; Mohamad Hajaj; Robert Dickens

#### InHealth Jarvis

##### ***Correspondence***: Kerrie Power

***Breast Cancer Research*** 2023, **25 (Suppl 2)**:P43

**Background:** Recall for breast screening assessment can be associated with significant client anxiety (1, p. 8). This anxiety can be further increased by undergoing biopsy. As with any interventional procedure, the risks include but are not limited to (2):

- Pain
- Haematoma formation
- Infection

This study aims to determine if multiple biopsies for potentially abnormal calcifications can be reduced, whilst maintaining high cancer detection. If possible, this would reduce unnecessary biopsy and client anxiety, as well as alleviate the workload pressure on the breast screening service.

The NHSBSP (3, p. 28) recommends that practice should be reviewed and that groups of interest are the last 20 clients who have had more than one biopsy procedure and also the last 20 clients who have had a benign result.

**Methods:** This retrospective study will investigate screening clients assessed between August 2021 and August 2022 who have undergone multiple x-ray guided biopsies for calcifications. For improved representativeness, a year’s worth of data was selected rather than the last 20 clients. The period selected is contemporary enough to be relevant but allows for final results for clients who have gone on to have further treatment.

A mixture of data regarding recall to assessment, biopsy method, results and MDM discussion is being gathered from MDM notes, assessment paperwork, NBSS and PACS.

No ethics considerations are required as no client identifiers will be used.

## Results:

Results will be available by May 2023.

**References**

1. NHSBSP. Clinical Guidance for Breast Cancer Screening Assessment. [Internet]. 2016 [cited 10 Jan 2023]. Available from: Clinical guidance for breast cancer screening assessment (publishing.service.gov.uk)
2. Cancer Research UK. Vacuum assisted biopsy. [Internet] 2020. [cited 10 Jan 2023]. Available from: https://www.cancerresearchuk.org/about-cancer/breast-cancer/getting-diagnosed/tests-diagnose/vacuum-assisted-biopsy
3. Quality Assurance Guidelines for Breast Cancer Screening Radiology. [Internet.] 2011 [cited 10 Jan 2023]. Available from: Colposcopy and Programme Management (publishing.service.gov.uk)

## P44 Is there a relationship between breast density and interval cancers? A clinical audit

### Kirsty Whitaker; Emily Nightingale

#### Rotherham NHS Foundation Trust

##### ***Correspondence***: Emily Nightingale

***Breast Cancer Research*** 2023, **25 (Suppl 2)**:P44

**Background:** Interval cancers, cancers diagnosed between screening rounds, are associated with poorer outcomes than screen detected cancers.^1,2^Interval cancers are commonly diagnosed in patients with greater breast density.^2^ These patients are at greater risk of developing cancers; there is also the potential for dense breast tissue to mask cancers on mammograms.^2^

Breast density is an international talking point for patients, policy-makers, and staff, particularly in the USA with many states requiring women to be notified regarding their breast density.^3^ In response to this and more patients querying their breast density at our Trust, we were inspired to undertake this audit.

**Method:** A retrospective audit of interval cancers diagnosed between 1st April 2017 and 31st March 2020 was completed.

113 interval cancers were identified using CREGX Cancer Registry Extract data on NBSS.

Cases were reviewed by a Reporting Radiographer and categorised as ‘dense’, ‘mixed’, or ‘fatty’ breast tissue.

Interval cancers were reviewed and graded as either category 1 (normal/benign)^4^, category 2 (difficult to perceive)^4^, category 3 (obviously malignant)^4^ by consultants.

**Results:** Of the 113 interval cancers identified:

- 95/113 (84%) had mixed or dense breast tissue.
- 74/89 (83%) graded category 1 had mixed/dense breast tissue.
- 21/24 (87.5%) graded category 2 had mixed/dense breast tissue.

**Conclusions:** From the cases reviewed a significant percentage had mixed/dense breast tissue, with only 16% of interval cancers having fatty breast tissue. The majority of category 2 interval cancers had mixed/dense breast tissue, which could potentially have masked a subtle cancer on the screening mammogram.

**References**

1. Strand, F. Azavedo, E. Hellgren, R. et al. (2019) *Localized mammographic density is associated with interval cancer and large breast cancer: a nested case–control study.* Available at: 10.1186/s13058-019-1099-y (Accessed: 25th July 2022).
2. Nguyen, T. L. Li, S. Dite, G. S. et al*.* (2020) ‘Interval breast cancer risk associations with breast density, family history and breast tissue aging’, *International Journal of Cancer*, 147 (2), pp. 375–382. Available at: https://onlinelibrary.wiley.com/doi/full/10.1002/ijc.32731 (Accessed: 25th July 2022).
3. Guterbock, T. M. Cohn, W. F. Rexrode, D. L. et al*.* (2017) *‘What Do Women Know About Breast Density? Results From a Population Survey of Virginia Women’*. Journal of the American College of Radiology, 14 (1), pp. 34–44. Available at https://www.sciencedirect.com/science/article/pii/S154614401630583X (Accessed 26th July 2022).
4. Public Health England. Breast Screening: reporting, classification and monitoring of interval cancers and cancers following previous assessment [Internet]. GOV.UK; 2021 [updated 2021 February 25; cited 2022 November 25]. Available from: https://www.gov.uk/government/publications/breast-screening-interval-cancers/breast-screening-reporting-classification-and-monitoring-of-interval-cancers-and-cancers-following-previous-assessment

## P45 Implementing tomosynthesis for routing breast cancer screening in England—cost implications of equipment upgrade/replacement

### Huajie Jin^1^; Kang Wang^1^; John Loveland^2^; Alistair Mackenzie^2^; Stephen Duffy^3^; Michael Michell^4^

#### ^1^King's College London; ^2^Royal Surrey NHSn Trust; ^3^Queen Mary University of London; ^4^King's College Hospital NHS Foundation Trust

##### ***Correspondence***: Kang Wang

***Breast Cancer Research*** 2023, **25 (Suppl 2)**:P45

**Background:** Compared with the current standard screening test for breast cancer (2D digital mammography (2DDM)), digital breast tomosynthesis (DBT) is more accurate but might be associated with increased cost. This study aims to assess the cost implications of equipment upgrade/replacement if DBT was used for routine breast cancer screening in England.

**Methods:** The 10-year cost implications of equipment upgrade/replacement was assessed for two different strategies: (1) No Switch (continue with 2DDM); (2) Gradually Switch (upgrade upgradable screening machines to DBT in Year 2023 and replace unupgradable machines when they expire). Due to a lack of data and resource constrains, only the cost of purchasing/upgrading the machines were included in this analysis. Information about the current screening machines (e.g., number, function, and age) were obtained from the NHSBSP equipment database. The cost of purchase and upgrade of the screening machine were obtained from the NHS Supply Chain.

**Results:** To implement DBT for all routine breast cancer screening in England, 53 screening machines need be upgraded and 210 need to be replaced as they are unupgradable. The average age of existing screening machines is 5.4 years (range: 1–13 years). The discounted 10-year cost of ‘No Switch’ and ‘Gradually Switch’ is £45.7 million and £48.2 million, respectively.

**Conclusion:** Switching to DBT will increase the cost of equipment upgrade/replacement by 2.6 million; but the increased budget could be potentially offset by savings in diagnostic tests and treatment. Our results can be updated when the long-term cost impacts of using DBT become available.

## P46 Siemens' intelligent optimum compression (Opcomp) function—a BHSCT review

### Paula Hughes; Michelle Murphy; Christine Greene; Jessica Robinson; Noelle Clerkin

#### Belfast Health and Social Care Trust

##### ***Correspondence***: Paula Hughes

***Breast Cancer Research*** 2023, **25 (Suppl 2)**:P46

**Background:** Compression is essential to optimum image quality, as it reduces image motion, geometric blur and differentiates between superimposed tissue and breast lesions, whilst reducing patient dose (1,2). Siemens’ Intelligent Optimum compression (Opcomp) function highlights to the Radiographer at the point of proposed optimal compression. This study aims to identify what is the average optimal compression point and if the function coincides with the mammographer’s applied compression selection.

**Methods:** In one screening session using three Siemens Mammo Inspirations, 61 clients were imaged.

Four trained Mammographers recorded the Newton force applied when undertaking a Right Medial Lateral Oblique (MLO) projection. The compression was also recorded at the point when the Opcomp feature was displayed.

**Results:** Opcomp feature displayed in 62% of mammograms performed. The mean newton value for the Opcomp to present was at 79N. Final Compression values applied after the Opcomp prompt delivered a variety of results from a reduction of 26N to an increase of 35N. On average the Radiographer applied 8.1N after the Opcomp feature was displayed.

**Conclusion:** Compression applied by 38% of cases did not meet the manufactures suggested optimum compression point. There is a paucity of information available on the Opcomp feature and how it optimises image quality in practice. In order to set benchmarks and utilise this feature, further investigation is required.

**References**

1. Feder, K. and Grunert, J.H., 2016. Is Individualizing Breast Compression during Mammography useful?-Investigations of pain indications during mammography relating to compression force and surface area of the compressed breast. RoFo: Fortschritte auf dem Gebiete der Rontgenstrahlen und der Nuklearmedizin, 189(1), pp.39–48.
2. Liu, Y.L., Liu, P.Y., Huang, M.L., Hsu, J.T., Han, R.P. and Wu, J., 2017. Simulation of breast compression in mammography using finite element analysis: A preliminary study. *Radiation Physics and Chemistry*, *140*, pp.295–299.

## P47 Does the addition of digital breast tomosynthesis within a departments breast imaging setting contribute to the patients pathway and reduce the benign biopsy rate?

### Ann Williams

#### Worcester Acute NHS Trust

##### ***Correspondence***: Ann Williams

***Breast Cancer Research*** 2023, **25 (Suppl 2)**:P47

**Objective:** Standard 2D spot compression and magnification are utilised in the workup of any suspicious M3 or M4 lesions seen on imaging. Breast Tomosynthesis was introduced within our service in 2021 to aid with the assessment of these equivocal lesions. This retrospective service evaluation aimed to establish if by the introduction of Digital Breast Tomosynthesis the patients journey can be improved and the benign biopsy rate reduced.

**Method:** 121 Indeterminate M3 and M4 lesions seen on 2D mammography diagnosed during the 6-month evaluation window on both symptomatic and screening breast patients were retrospectively analysed alongside the subsequent Digital Breast Tomosynthesis views. Data collection included: 2D and 3D image grading, lesion size and lesion features. Data was correlated with image guided biopsy results and surgical outcomes when undertaken. As patients were not able to be followed up as part of the evaluation the histological outcome was deemed the gold standard for analysis. This data was then analysed used both descriptive and inferential analysis to investigate the relationship between the various data recorded.

**Results:** The evaluation of 121 consecutive patients found that there was a significant reduction in M3 grading on 2D mammography with the addition of the 3D DBT. Out of the 121 patients that were subjected to 3D DBT: 51 were graded as normal, 25 were proven benign at biopsy, 4 were graded as B3 requiring surgery and 41 were proven malignant. In total following DBT only 70 patients went on to have a biopsy reducing the biopsy rate by 42.15%. Features identified on 2D mammography included 60 masses, 29 distortions, 31 asymmetric densities and 1 lymph node. The true positive rate was increased from 3% with 2D imaging to 26% with the combined imaging.

**Conclusion:** The addition of Digital Breast Tomosynthesis in the unit for the assessment of equivocal M3 and M4 lesions reduced the benign biopsy rate, and aided in the shortening time to diagnosis without compromising the detection of breast cancer. This study highlighted the important of incorporating new imaging technologies into clinical practice to improve patient care and outcomes.

**Advances in knowledge:** This evaluation has identified the added benefit to a breast imaging service with the addition of 3D DBT. The ongoing use of the modality can reduce the burden on the pathologist’s workload by reducing the benign biopsy rate and improve the patients physical and psychological experience.

## P48 The use of the mammography chair in reducing work related musculoskeletal disorders

### Iram Shabana

#### Nottingham University Hospitals

##### ***Correspondence***: Iram Shabana

***Breast Cancer Research*** 2023, **25 (Suppl 2)**:P48

**Background:** Whilst mammography technology is constantly improving, the technique has remained the same. Although, it is successful in acquiring diagnostic images for breast disease diagnosis, it comes with some difficulties for Mammographers.

Conventional mammography technique requires the mammographer to move into awkward and strenuous positions. Repetitive movements adopted by the mammographer have been associated with work related musculoskeletal disorders (WRMSDs) (1), resulting in long term sickness. In this current climate with a shortage of mammographers, we need to ensure that we are looking after our staff’s wellbeing.

Public Health England’s 2018 publication, ‘Breast screening mammography: ergonomics good practice’ provides guidance for mammographic staff to adopt best practice to avoid or minimise harm from work related injuries (WRIs) (2).

**Methods:** Difference in stature between the mammographer and patient is one major factor that can cause complications during the procedure which can lead to WRMSDs (3).

This poster will use effective illustrations with annotations to demonstrate the benefits of using the mammography chair to sit patients down whilst performing CC views, allowing for the reduction in WRMSDs. This method works well when using a specialised chair, solely designed to assist in performing mammograms (4).

**Conclusion:** This poster aims to showcase the benefits of the mammography chair especially for mammographers of shorter stature. It can be used as an educational tool to identify and communicate an improvement in mammography technique. Subsequently, allowing for a change to help avoid or minimise WRIs within mammographic staff, providing a solution to support the government’s guidance (2).

**References**

1. Borrelli CD. Repetitive strain injury – RSI. In: Hogg P, Kelly J, Mercer C, editors. Digital Mammography. Switzerland: Springer; 2015. 195–202.
2. Cernean N, Serranheira F, Gonçalves P, Sá Dos Reis C. Ergonomic strategies to improve radiographers' posture during mammography activities. Insights Imaging. 2017 Aug;8(4):429–438. 10.1007/s13244-017-0560-7. Epub 2017 Jun 21. PMID: 28639113; PMCID: PMC5519499.
3. England PH. Breast screening: Ergonomics in screening mammography [Internet]. GOV.UK. 2018Oct22 [cited 2023Jan13]. Available from: https://www.gov.uk/government/publications/breast-screening-ergonomics-in-screening-mammography
4. Vela Medical. Vela mammography chair—best conditions for effective examinations [Internet]. VELA Medical. 2022 [cited 2023Jan15]. Available from: https://vela-medical.com/chair/vela-mammography-chair/

## P49 Keeping abreast with breast cosmetic surgery and its myriad of appearances on multi-modality imaging

### Yee Ting Sim; Konstantia Stavrou; Leila Ismail; Alexandra Economacos

#### Mediclinic City Hospital & Comprehensive Cancer Centre, Dubai, UAE

##### ***Correspondence***: Yee Ting Sim

***Breast Cancer Research*** 2023, **25 (Suppl 2)**:P49

**Background**: We encounter increasing number and variety of breast cosmetic augmentation surgery being offered commercially and conducted on patients, who often have little information as to the technique or material utilised.

**Methods**: Patients with history of breast cosmetic surgery, including implant augmentation, filler injections, fat transfer, are collated on our database. We reviewed their imaging and selected cases with key learning points, particularly patients who underwent non-conventional imaging techniques such as contrast-enhanced mammography.

**Results:** Through this pictoral review, we present the spectrum of imaging appearances of the different types of breast augmentation surgery on conventional mammography, ultrasound, MRI through to the newer contrast-enhanced spectral mammography (CESM). Patients who had undergone free silicone injections pose significant challenge in breast screening evaluation; we have used MRI and CESM as alternatives, but these did not achieve additional improvement in diagnostic image quality.

**Conclusions**: Knowledge of normal radiologic features of the range of breast cosmetic surgery and their common complications is useful in the accurate diagnosis and evaluation of breast conditions.

**References**

- Raj SD, Karimova EJ, Fishman MDC et al. Imaging of breast implant-associated complications and pathologic conditions: breast imaging. Radiographics 2017; 37 (5): 1603–1604.
- Harvey KL, Clark SE. A guide to breast implants for the non-breast specialist. Womens Health (Lond). 2016 Nov;12(6):533–537. 10.1177/1745505716687562. Epub 2017 Feb 10. PMID: 29334026; PMCID: PMC5373262.

## P50 Imaging features of breast abscesses and other inflammatory breast disorders: A pictorial review

### Sharon Koo; Tom Hughes; Sarah Mcwilliams

#### St George's University Hospital

##### ***Correspondence***: Sarah Mcwilliams

***Breast Cancer Research*** 2023, **25 (Suppl 2)**:P50

Imaging features of breast abscesses, granulomatous mastitis, and inflammatory breast carcinoma will be presented and clinical manifestations, differential diagnoses, and management discussed. Various percutaneous drainage options including use of vacuum drainage will be discussed.

Breast infections and abscesses are a common presentation to the symptomatic clinic. Radiology plays a pivotal role in their diagnoses and management, particularly owing to the move away from conventional approaches of surgical incision and drainage, to more minimally invasive image-guided procedures. Ultrasound is vital in identifying the presence and size of abscesses, guiding radiological intervention, and monitoring progress. Imaging features of infective abscesses can overlap with other more sinister aetiologies such as granulomatous disorders or inflammatory breast carcinoma, making diagnoses challenging. Awareness of their clinical and radiological features is crucial.

Breast abscesses can take on a long clinical course and warrant close monitoring. Radiologists play an essential role in evaluation, follow up and treatment. Prompt drainage reduces the risk of multifocal abscesses and need for surgical intervention, which is challenging in many of these postpartum women. Recognition of inflammatory breast carcinoma is crucial in patients with poor response to antibiotic therapy and warrants biopsy.

## P51 A rare cause of breast calcifications

### Arlene Weir^1^; Lorna Duddy^2^; Alissa Connors^2^

#### ^1^BreastCheck Southern Unit/Cork University Hospital; ^2^BreastCheck Southern Unit

##### ***Correspondence***: Arlene Weir

***Breast Cancer Research*** 2023, **25 (Suppl 2)**:P51

**Background:** Breast calcifications are tiny flecks of calcium which are commonly seen on mammography, which have various causes, however ductal carcinoma-in-situ represents 25–30% of all reported breast cancers and 95% of all DCIS is diagnosed because of mammographically detected microcalcifications (1). Accurate evaluation of calcifications is vitally important and characterisation based on morphological features alone may be challenging, with tomosynthesis or stereotatic biopsy frequently being performed. The majority of benign calcifications are due to fibroadenomatoid or fibrocystic change, however there are also other rare and unusual causes.

**Material and methods:** We retrospectively reviewed the imaging findings and histopathology results of two patients with breast calcifications.

**Results:** Two patients with indeterminate breast calcifications on mammography without a discrete mass subsequently had tomosynthesis guided biopsies. Histopathology appearances were initially also indeterminate, however further evaluation with congo red staining showed apple green birefrigence under polarized light in keeping with a diagnosis of breast amyloid.

**Conclusions:** Breast amyloid can be either primary or secondary and is a rare cause of breast calcifications with the first case reported in 1973. Radiographic findings vary but appearances may mimic DCIS or invasive ductal carcinoma. Breast amyloid occurs in < 1% of people with amyloidosis, however 25% of breast amyloid is associated with systemic disease. Primary breast amyloid does not normally progress to systemic disease, however 50% of cases are associated with a haematological disease such as lymphoma and patient's should be referred for haematological work-up.

**Reference**

1. Smithuis R, Pijnappel R. Differential of breast calcifications [Internet]. The Radiology Assistant: Differential of Breast Calcifications. Radiology department, Rijnland Hospital, Leiderdorp and Martini Ziekenhuis, Groningen, the Netherlands.; [cited 2023Jan15]. Available from: https://radiologyassistant.nl/breast/calcifications/differential-of-breast-calcifications

## P52 Male breast diseases: A pictorial overview at a local NHS Trust

### Juliet Mazarura

#### University Hospitals Birmingham NHS Trust

##### ***Correspondence***: Juliet Mazarura

***Breast Cancer Research*** 2023, **25 (Suppl 2)**:P52

**Background:** Male breast cancer is rare (comprising less than 1% of all breast cancers) whereas gynaecomastia is very common (A Shaaban 2019).

However, there is a range of other male breast disease which is encountered in breast imaging departments. Male breast disease can be distressing for the patient and their relatives with feelings of emasculation, anxiety, depression and embarrassment due to their condition (Kipling et al. 2014).

## Aim:

1. To raise awareness of common male breast disease, despite different local imaging protocols.
2. To illustrate radiological features of common breast disease in a pictorial poster so as to ensure effective patient management.

**Breast pathology includes**: Breast Cancer, DCIS, Gynecomastia, Lipoma/fat necrosis

Benign breast disease (papilloma, sebaceous cysts, haematoma fibroepithelial lesions and abscess (Williams & Metelko 2019.)

**Conclusion:** Breast imaging practitioners need to be aware of common radiological male breast disease features. Men should be offered a service tailored to their breast imaging needs.

**References**

1. Abeer M.Shaaban Pathology of the male Diagnostic Histopathology Volume 25, Issue 4, April 2019, Pages 138–142 https://www.sciencedirect.com/journal/diagnostic-histopathology
2. Best practice diagnostic guidelines for patients presenting with breast symptoms. NICE Nov 2010 https://www.evidence.nhs.uk/document?id=2013590&returnUrl=search%3Fq%3Dhc11%26sp%3Don&q=hc11
3. Mike Kipling 1, Jane E M Ralph 1, Keith Callanan 1Psychological impact of male breast disorders: literature review and survey results Breast Care (Basel) 2014 Feb;9(1):29–33.10.1159/000358751.
4. Male breast ultrasound: 2019 audit results Umit Aksoy Ozcan, Susan Williams, Marie Metelko

